# Family with sequence similarity 114 member A1 orchestrates immune evasion in triple-negative breast cancer

**DOI:** 10.1038/s41392-025-02472-9

**Published:** 2025-11-18

**Authors:** Wenhao Zhang, Yanzhi Gai, Mengxue Qiao, Michelle Rowicki, Yong Wei, Xiang Hang, Zhengkai Wei, He Yang, Xifu Ye, Hang Ju, Yi Lu, Yibin Kang, Minhong Shen

**Affiliations:** 1 Key Laboratory of Breast Cancer in Shanghai, Department of Breast Surgery, Fudan University Shanghai Cancer Center, Shanghai, China; 2 Tongji University Cancer Center, Shanghai Tenth People’s Hospital, School of Medicine, Tongji University, Shanghai, China; 3 Department of Molecular Biology, Princeton University, Princeton, NJ USA; 4 Cancer Metabolism and Growth Program, The Cancer Institute of New Jersey, New Brunswick, NJ USA; 5 Ludwig Institute for Cancer Research Princeton Branch, Princeton, NJ USA; 6 Department of Oncology, Shanghai Medical College, Fudan University, Shanghai, China

**Keywords:** Breast cancer, Cancer microenvironment

## Abstract

Immune checkpoint blockade (ICB) therapy, which has revolutionized cancer treatment, has been approved for the treatment of triple-negative breast cancer (TNBC). Unfortunately, most patients with TNBC are either not eligible for treatment or exhibit resistance, resulting in limited overall survival benefits. There is an urgent need to elucidate the mechanisms of resistance and enhance therapeutic efficacy. Here, via CRISPR activation (CRISPRa) screening, we identified *family with sequence similarity 114 member A1* (*FAM114A1*) as a key mediator of immune evasion and ICB resistance in TNBC. Mechanistically, FAM114A1 binds p85α to disrupt the p85α/p110α protein complex, thus activating the PI3K/AKT pathway and simultaneously preventing condensate formation of E2F Transcription Factor 4 (E2F4) to promote E2F4-driven Metadherin (MTDH) transcription. Upregulation of these FAM114A1-mediated pathways suppresses tumor antigen presentation and consequently attenuates antitumor immunity in TNBC. Moreover, targeting FAM114A1 improves the therapeutic effectiveness of anti-PD-1 therapy in mouse models, and a FAM114A1-based signature shows strong predictive performance for identifying patients with TNBC who may benefit from ICB. Collectively, our findings not only reveal that FAM114A1 is an immune evasion driver but also highlight it as a promising biomarker and therapeutic target. Our study provides new insights into TNBC immune evasion and outlines a potential avenue to improve the effectiveness of ICB.

## Introduction

Triple-negative breast cancer (TNBC), which accounts for 10‒20% of all breast cancer cases, is an aggressive molecular subtype characterized by the absence of estrogen receptor (ER) and progesterone receptor (PR) coupled with low human epidermal growth factor receptor 2 (HER2) protein expression.^1^ This unique molecular profile has hindered the development of targeted therapies for TNBC. It has led to TNBC being classified as a subtype with poor patient prognosis. Immune checkpoint blockade (ICB), which reactivates antitumor immunity, has transformed cancer treatment and achieved considerable success in multiple malignancies.^2^ Studies in melanoma and non-small cell lung cancer patients have established ICBs, particularly the anti-PD-1/PD-L1 agents, as standard-of-care agents, with landmark trials revealing durable responses and improved overall survival in subsets of patients with high tumor mutational burdens or PD-L1 expression.^3,4^ ICB strategies have also been approved for the treatment of TNBC.^5^ Unfortunately, resistance arises in many patients, which significantly hinders the therapeutic efficacy of ICB.^6,7^ There is an urgent need to identify resistance mechanisms and target key effectors to increase the effectiveness of ICB in TNBC patients.

CRISPR-Cas9 screening has emerged as a robust, high-throughput methodology for systematically identifying genetic determinants of immune evasion and immunotherapy resistance in cancer.^8^ In contrast to conventional CRISPR-Cas9 systems that mediate gene knockout via DNA double-strand breaks, modified platforms employing catalytically inactive Cas9 (dCas9) have been developed for gain-of-function screens. When coupled to transcriptional activation domains (e.g., VP64), the dCas9 fusion protein enables precise transcriptional activation of target‒gene transcription, a technology termed CRISPR activation (CRISPRa).^9^ This innovative approach enables genome-wide identification of phenotypic drivers by inducing gene overexpression rather than disrupting gene function. Importantly, CRISPRa screens specifically pinpoint genes whose elevated expression is sufficient to elicit defined biological outcomes, such as antitumor immunity regulation.^10^ However, to comprehensively understand the mechanisms underlying tumor immune evasion, we not only need to identify the genes that are involved in this process but also have to dissect the following detailed signal transduction cascades. Recent studies demonstrated that proteins condense into liquid-like droplets to promote psychological and pathological processes.^11–13^ For instance, KAT8-IRF1 was found to form liquid-like condensates to enhance PD-L1 expression, and thus, to suppress antitumor immunity.^14^ It suggests that blocking liquid-like condensate formation may serve as a therapeutic strategy for cancer immune therapy. Supporting this notion, attempts have been made to enhance drug efficacy by taking advantage of liquid-like condensates.^15^ Although new perspectives for understanding antitumor immunity are arising upon exploring liquid-like condensates, we are still in its infancy stage, and more studies are needed to comprehensively understand the mechanisms underlying liquid-like condensate formation and the consequent antitumor immune responses.

PI3K/AKT hyperactivation, which contributes to cancer progression and treatment resistance,^16^ have been observed in breast cancer patients.^17^ Clinical trials that target the PI3K/AKT pathway have achieved considerable success, and this therapeutic strategy has been approved for treating hormone receptor-positive advanced breast cancer patients.^18–20^ PI3K/AKT activation is also critical for TNBC progression.^21,22^ Preclinical studies indicated that PI3K/AKT inhibition could possibly sensitize TNBC to ICB.^23,24^ Unfortunately, a clinical trial that combined PI3K/AKT inhibition and ICB treatment resulted in unsatisfactory responses in TNBC patients. The randomized phase III IPATunity170 trial (NCT04177108) indicated that adding ipatasertib, which is an ATK inhibitor, did not significantly enhance the progression-free survival and overall survival compared with atezolizumab plus paclitaxel in TNBC patients with PD-L1-positive.^25^ Interestingly, in the PD-L1-negative/unknown TNBC cohort, median progression-free survival was improved from 5.6 months to 7.1 months after adding atezolizumab to ipatasertib plus paclitaxel treatment; however, this difference has not been observed for overall survival.^25^ Collectively, it suggests that the expression of PD-L1 may not be a good biomarker to stratify TNBC patients who may benefit from ICB treatment. More importantly, PI3K/AKT inhibition is not sufficient to sensitize TNBC patients to ICB therapy, suggesting other pathways could compensate for PI3K/AKT inhibition to result in immunotherapy resistance. Identifying better biomarkers to distinguish ICB responders and non-responders and dissecting pathways that compensate PI3K/AKT inhibition could enhance ICB treatment efficacy in TNBC patients.

In this study, we performed a CRISPRa screen to identify candidate genes that could confer resistance to ICB in TNBC. The *family with sequence similarity 114 member A1* (*Fam114a1*) emerged as a top-ranked candidate, and its immune evasion-promoting role was validated in both in vitro and in vivo models. Mechanistic characterization demonstrated that FAM114A1 activated the PI3K/AKT pathway and simultaneously prevented the formation of liquid-like E2F Transcription Factor 4 (E2F4) condensates, thereby enhancing E2F4-mediated transcriptional activation of Metadherin (MTDH). Both pathways converge to impair the antigen presentation machinery, thereby facilitating immune escape. Notably, FAM114A1-targeting sensitizes TNBC to anti-PD-1 treatment in mouse models, suggesting that FAM114A1 may serve as a therapeutic target. On the basis of these findings, we developed a FAM114A1-centric molecular signature that showed robust performance in identifying patients with TNBC who may benefit from ICB.

## Results

### FAM114A1 facilitates TNBC immune evasion

**Fig. 1:**
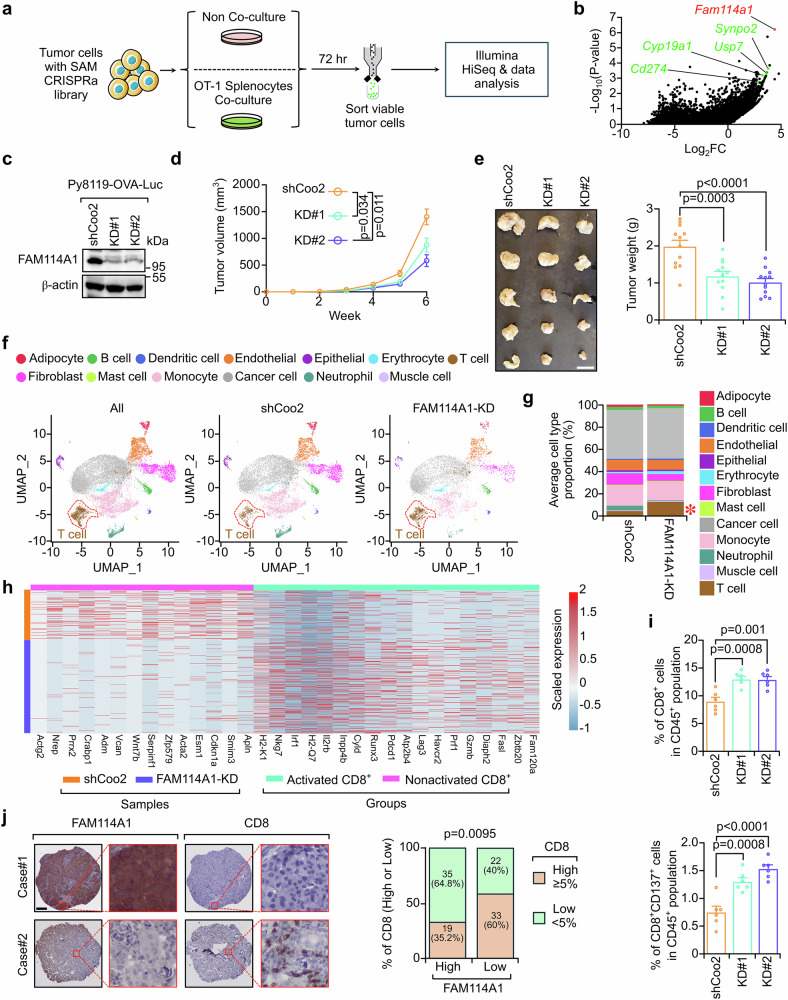
FAM114A1 promotes TNBC immune evasion. **a** Schematic diagram illustrating tumor/immune coculture-based CRISPRa screening used to identify genes that promote immune evasion. Py8119-OVA-dCas9-P65/HSF1 cells were used for screening. **b** The enrichment status of genes was plotted in comparison with that of the non-coculture group. **c** Py8119 cells labeled with ovalbumin and luciferase (Py8119-OVA-Luc) were used to generate stable endogenous FAM114A1-knockdown cell lines. Cells with stable FAM114A1-knockdown (KD#1 and KD#2) and the corresponding control (shCoo2) were collected for western blotting to test the efficacy of FAM114A1-knockdown. **d**, **e** Py8119-OVA-Luc cells with and without FAM114A1-knockdown were injected into the mammary fat pads of female OT-I mice. Tumor growth was monitored and measured weekly (**d**). The tumors were dissected, and tumor weight was measured at the endpoint (**e**). Representative tumors are shown (**e**, left panel). n = 12 mice per group. Bar, 1 cm. **f** Tumors from the above groups were collected, formalin fixed, and paraffin embedded (FFPE). FFPE tumors were subjected to single-cell RNA sequencing. UMAP plots of the cell populations from the indicated groups. The T cells in each group are highlighted with red circles. **g** Average cell type proportions in the control (shCoo2) and FAM114A1-knockdown groups (FAM114A1-KD). The red start indicates the T-cell population. **h** Heatmap showing activated and nonactivated marker genes expressed in CD8^+^ T cells from the shCoo2 and FAM114A1-KD groups. **i** Fresh tumors from **d** were collected for flow cytometry analysis. The percentages of the indicated populations are shown. shCoo2, control group; KD#1 and KD#2, tumors with endogenous FAM114A1-knockdown. n = 6 tumors per group. **j** FFPE samples from TNBC patients were subjected to immunohistochemistry (IHC) staining with anti-FAM114A1 or anti-CD8 antibodies. Representative images are shown. The expression levels of FAM114A1 were quantified with ImageJ, and positive ratios of CD8^+^ T cells were determined. High- and low-FAM114A1 expression was determined by the median expression. Samples with ≥5% CD8^+^ T-cell infiltration were defined as CD8^+^ T-cell high, and those with <5% infiltration were defined as CD8^+^ T-cell low^.^ n = 109 TNBC patients. Bar, 500 µm. The data represent the means ± SEMs. P-values were determined by one-way ANOVA (**d**, **e**, **i**) or the χ^2^ test (**j**)

To identify genes that potentially suppress immunosurveillance in TNBC, we employed a tumor/immune cell cocultured-based CRISPR activation (CRISPRa) screen. As described in our previous study,^26^ we developed an ovalbumin (OVA)/OT-I-mediated tumor/immune in vitro coculture system in which OVA-expressing tumor cells are specifically recognized and eliminated by immune cells isolated from OT-I mice. We then incorporated a CRISPRa screening platform^27^ into this coculture system to identify genes that enhance immune evasion. To this end, CRISPRa screening components were first transduced into the mouse TNBC cell line Py8119^28^ by using lentivirus to generate Py8119-OVA-dCas9-P65/HSF1 cells (Supplementary Fig. 1a). Stable expression of these components did not alter OVA antigen presentation or the consequent immune-mediated killing (Supplementary Fig. 1b, c). Next, we transduced a genome-scale mouse CRISPRa library^27^ and cocultured the cells with splenocytes isolated from OT-I mice (Fig. 1a and Supplementary Fig. 1d). The vast majority of the tumor cells were eliminated after 72 h of coculture. Viable tumor cells were sorted, non-cocultured tumor cells served as a negative control, and enriched genes were identified via next-generation sequencing (Fig. 1a, b). The *family with sequence similarity 114 member A1* (*Fam114a1*) was among the most enriched genes (Fig. 1b), suggesting that FAM114A1 overexpression may promote TNBC immune evasion. As expected, we also observed the enrichment of genes previously implicated in immune evasion, such as *Synpo2*,^29,30^ *Usp7*,^31^ *Cyp19a1*,^32^ and *Cd274*.^33^ These findings support the robustness of this screening platform in identifying genes that enhance immune evasion. To confirm that FAM114A1 was overexpressed in our CRISPRa system, three guide RNAs targeting FAM114A1 (Supplementary Fig. 1e) were cloned and inserted into the same backbone used for screening.^27^ The three guide RNAs were pooled and transduced into Py8119-OVA-dCas9-P65/HSF1 cells. The cells were then harvested for western blotting to assess FAM114A1 expression. Compared with control cells, cells transduced with FAM114A1-targeting guide RNAs presented higher FAM114A1 protein levels (Supplementary Fig. 1f), confirming the effective upregulation of FAM114A1 by our CRISPRa platform. Next, we asked whether FAM114A1 promotes immune evasion in TNBC. To this end, we stably knocked down this gene in Py8119 cells labeled with OVA and firefly luciferase (Py8119-OVA-Luc) to enable tumor/immune coculture assays (Fig. 1c). FAM114A1-knockdown (KD hereafter) significantly increased immune killing (Supplementary Fig. 1g). To further assess the functional importance of FAM114A1 in vivo, tumor cells with and without FAM114A1-KD were orthotopically injected into OT-I female mice. These results support the conclusion that FAM114A1-KD inhibits TNBC progression (Fig. 1d, e).

Next, formalin-fixed paraffin-embedded (FFPE) tumors with or without FAM114A1-KD were subjected to Flex single-cell RNA sequencing (scFFPE-Seq) to investigate how FAM114A1 promotes immune evasion. As expected, FAM114A1-KD tumors contained significantly fewer tumor cells with detectable FAM114A1 (Supplementary Fig. 2a), indicating the robustness of FAM114A1-KD at the time of harvest. Compared with control tumors, FAM114A1-KD tumors contained more T cells (Fig. 1f, g), including CD8^+^ T cells (Supplementary Fig. 2b). Furthermore, CD8^+^ T cells collected from FAM114A1-KD tumors presented increased expression of activation-associated genes (Fig. 1h). To corroborate the scFFPE-Seq findings, we performed immunohistochemistry (IHC) and flow cytometry analyses, which again revealed increased CD8^+^ T-cell infiltration and activation in FAM114A1-KD tumors (Fig. 1i and Supplementary Fig. 2c, d).

To investigate whether FAM114A1 inhibits CD8^+^ T-cell infiltration and activation in patients, we analyzed single-cell RNA sequencing (scRNA-Seq) data from a prior TNBC cohort (NCT03197389).^34^ Patients were stratified into high- and low-*FAM114A1* groups on the basis of median expression (Supplementary Table 1). Consistent with the murine data, tumors from *FAM114A1-*low patients harbored increased numbers of T cells, CD8^+^ T cells, and activated CD8^+^ T cells (Supplementary Fig. 3). To experimentally validate these observations, we performed IHC on FFPE tumor samples from 109 patients with TNBC (Supplementary Table 2) and found that higher FAM114A1 expression was associated with significantly less CD8^+^ T-cell infiltration (Fig. 1j). Collectively, our data suggest that FAM114A1, identified via CRISPRa screening, facilitates TNBC progression by suppressing antitumor immunity.

### FAM114A1 activates the PI3K/AKT pathway and suppresses antigen presentation

**Fig. 2:**
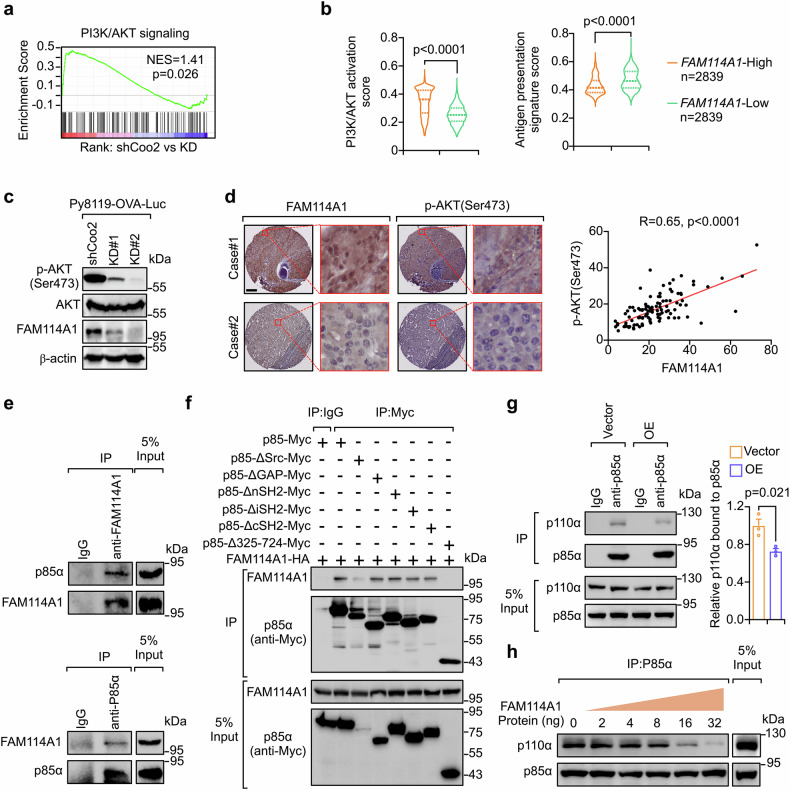
FAM114A1 activates the PI3K/AKT pathway and suppresses antigen presentation. **a** Expression profile of cancer cells extracted from scFFPE-Seq of shCoo2- and FAM114A1-KD tumors. Gene set enrichment analysis (GSEA) was performed, and PI3K/AKT pathway enrichment in the shCoo2 group is shown. **b** scRNA-seq data of cancer cells extracted from TNBC patients. Cells without *FAM114A1* expression were excluded, and the remaining cells were stratified into high- and low-FAM114A1 groups on the basis of median expression. PI3K/AKT activation scores (left panel) and antigen presentation signature scores (right panel) were calculated. **c** Py8119-OVA-Luc cell lines with (KD#1 and KD#2) and without (shCoo2) FAM114A1-knockdown were cocultured with OT-I splenocytes for 30 min. The tumor cells were isolated by sorting and subsequently collected for Western blotting. The expression levels of phosphorylated AKT (Ser473), total AKT, and FAM114A1 were tested. β-actin served as an internal control. **d** TNBC patient samples were analyzed by immunohistochemistry with anti-FAM114A1 and anti-phosphorylated AKT (Ser473) antibodies. Representative images are shown (left panel). The expression levels of FAM114A1 and phosphorylated AKT were quantified with ImageJ and analyzed with Spearman’s rank correlation (right panel). n = 109 TNBC patients. Bar, 500 µm. **e** Py8119 tumor cell lysates were collected and coimmunoprecipitated (co-IP) with anti-FAM114A1 (upper panel), anti-p85α (lower panel), or IgG controls. The interaction between p85α and FAM114A1 was determined via western blotting. **f** 293T cells were cotransfected with the indicated plasmids. Twenty-four hours after transfection, the cells were lysed and subjected to co-IP analysis. The samples were then analyzed via western blotting to detect interactions. **g** Py8119 cells stably expressing FAM114A1 were generated. The cells with (OE) or without (Vector) FAM114A1 overexpression were collected for co-IP analysis. The level of p110α that interacted with p85α was quantified after normalization to total p110α (right panel). **h** 293T cells were cotransfected with p85α and p110α plasmids. The cells were collected for co-IP after 24 h. The Co-IP samples from the beads were divided into 6 tubes and incubated with the indicated amount of the FAM114A1 recombinant protein for 2 h. The beads were washed thoroughly with lysis buffer, and the bound proteins were subjected to western blotting to detect p85α and p110α interactions. The data represent the means ± SEMs. P-values were determined by a two-tailed Student’s *t*-test (**b, g**)

To investigate the mechanisms underlying FAM114A1-mediated immune evasion, tumor cells from scFFPE-Seq were analyzed. Pathway enrichment analysis revealed that PI3K-related pathways were enriched in control cells, and antigen processing and presentation pathways were enriched in FAM114A1-KD tumor cells (Fig. 2a and Supplementary Fig. 4a, b), revealing that FAM114A1 may be involved in these pathways. Consistent with this finding, TNBC patients from the Fudan University Shanghai Cancer Center (FUSCC) cohort (SRP157974)^35^ (Supplementary Table 1), with high- and low-*FAM114A1* expression, presented enriched PI3K and antigen presentation pathways, respectively (Supplementary Fig. 4c), which is consistent with the finding of FAM114A1 control and KD tumors in mouse models. Similarly, scRNA-Seq data from TNBC patients (NCT03197389)^34^ indicated that tumor cells with high *FAM114A1* expression presented increased PI3K/AKT activation and reduced tumor antigen presentation (Fig. 2b). Taken together, these results suggest that FAM114A1 may activate the PI3K/AKT pathway and suppress antigen presentation signaling. Coculture experiments confirmed these sequencing data, showing that FAM114A1-KD reduced PI3K/AKT activation and increased antigen presentation (Fig. 2c and Supplementary Fig. 5a). In support of this hypothesis, IHC staining of TNBC patient samples revealed a positive correlation between FAM114A1 expression and phosphorylated AKT levels (Fig. 2d). Interestingly, we found that patient tumor cells (from the NCT03197389 cohort) with a higher PI3K/AKT activation signature presented lower antigen presentation (Supplementary Fig. 5b), which is consistent with previous findings that PI3K activation suppresses antigen presentation in epithelial cells.^36^ We also experimentally confirmed that PI3K inhibition enhanced antigen presentation in tumor cells (Supplementary Fig. 5c). Overall, these findings indicate that FAM114A1 may activate PI3K/AKT signaling to suppress tumor cell antigen presentation. As supported by previous studies, PI3K/AKT signaling suppresses antigen presentation by downregulating MHC-I and MHC-II gene expression^36^ and promoting the internalization of the MHC-I complex, thereby reducing antigen surface display.^37,38^

Next, we investigated how FAM114A1 activates PI3K/AKT signaling. To this end, we first performed coimmunoprecipitation (co-IP), followed by mass spectrometry, to identify FAM114A1-binding partners (Supplementary Fig. 5d and Supplementary Table 3). Pathway enrichment analysis of potential FAM114A1-binding partners identified by co-IP mass spectrometry further confirmed that FAM114A1 may be involved in PI3K/AKT and antigen presentation regulation (Supplementary Fig. 5e). Notably, among these identified proteins, p85α was one of the top candidates directly involved in PI3K/AKT signaling. Using co‑IP assays, we validated that FAM114A1 interacts with p85α (Fig. 2e and Supplementary Fig. 5f). Additionally, we mapped the interaction domain of p85α and found that triple depletion of the N-terminal, inter, and C-terminal Src homology 2 (nSH2, iSH2, and cSH2) domains completely abolished its binding to FAM114A1 (Fig. 2f and Supplementary Fig. 5g). Interestingly, depletion of these three domains prevented p85α from binding to p110α (Supplementary Fig. 5h), which is consistent with previous findings.^39,40^ Since FAM114A1 and p110α may bind to the same domains of p85α, we hypothesized that FAM114A1 disrupts the interaction between p85α and p110α. To test this hypothesis, we generated Py8119 cells stably overexpressing FAM114A1 (Supplementary Fig. 5i). The cells were then used for co-IP assays to examine the interactions between p85α and p110α. We found that FAM114A1 overexpression reduced p85α–p110α interactions in tumor cells (Fig. 2g). In line with this, the FAM114A1 recombinant protein disrupted the binding of p85α to p110α (Fig. 2h and Supplementary Fig. 5j). As a regulatory repressive subunit of PI3K, p85α binds to and inhibits p110α catalytic activity and downstream PI3K/AKT signaling.^41–44^ Our data, together with previous findings, suggest that FAM114A1 may disrupt the p85α/p110α complex to activate PI3K/AKT signaling, thereby suppressing antigen presentation in tumor cells.

### FAM114A1 prevents liquid-like condensate formation of E2F4

**Fig. 3:**
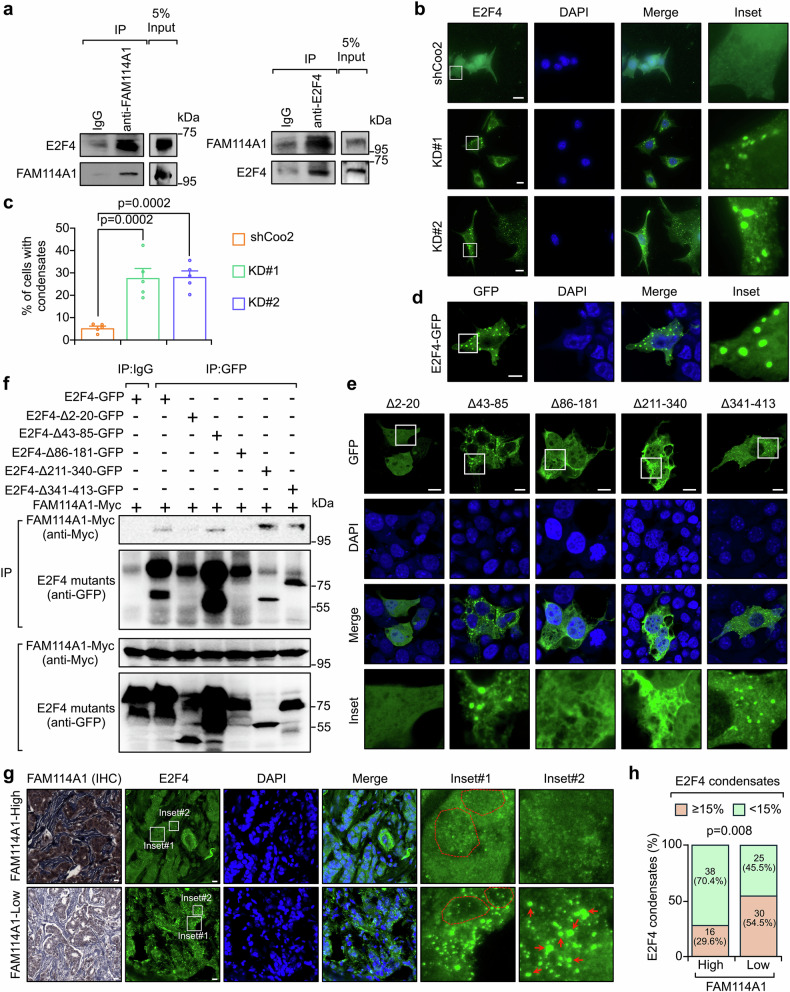
FAM114A1 binds E2F4 to inhibit its condensate formation. **a** Py8119 tumor cell lysates were collected and immunoprecipitated with anti-FAM114A1 (left panel), anti-E2F4 (right panel) antibodies, or IgG controls. The interaction between E2F4 and FAM114A1 was determined via western blotting. **b**, **c** Py8119 cells with (KD#1 and KD#2) and without (shCoo2) FAM114A1-knockdown were fixed and subjected to immunofluorescent (IF) staining with an anti-E2F4 antibody. DNA was visualized with DAPI (**b**). The percentage of cells with E2F4 condensates was quantified (**c**). Bar, 10 µm. **d** Py8119 cells were transfected with the E2F4-GFP plasmid. Twenty-four hours after transfection, the cells were fixed for confocal imaging. DNA was visualized with DAPI. Bar, 10 µm. **e** Py8119 cells were transfected with the indicated E2F4-GFP mutant plasmids. Twenty-four hours after transfection, the cells were fixed for confocal imaging. DNA was visualized with DAPI. Bar, 10 µm. **f** 293T cells were cotransfected with the FAM114A1-Myc plasmid together with the indicated E2F4-GFP mutants. The cells were collected for co-IP after 24 h. The samples were subjected to western blotting to determine interactions. **g** Samples from TNBC patients were subjected to IHC staining with an anti-FAM114A1 antibody or IF staining with an anti-E2F4 antibody. DNA was visualized with DAPI. The nuclear areas are highlighted with red dashed circles in inset #1. Representative E2F4 condensates are indicated by red arrows in inset #2. Bar, 10 µm. **h** FAM114A1 expression was quantified with ImageJ after IHC staining, and the samples were stratified into high- and low-FAM114A1 groups on the basis of median expression. The number of cells with E2F4 condensates was also quantified via IF staining. The data represent the means ± SEMs. P-values were determined via one-way ANOVA (**c**) or the χ^2^ test (**h**)

In addition to p85α, E2F4 was identified as the top interacting partner of FAM114A1 (Supplementary Fig. 5d and Supplementary Table 3). The interaction between FAM114A1 and E2F4 was confirmed by co-IP experiments (Fig. 3a and Supplementary Fig. 6a). To investigate whether E2F4 is also involved in FAM114A1-mediated tumor immune evasion, we extensively explored the role of E2F4 in this context. First, we found that E2F4 formed puncta in tumor cells and that the proportion of cells with puncta significantly increased upon FAM114A1-KD (Fig. 3b, c, and Supplementary Fig. 6b, c), suggesting that FAM114A1 inhibited E2F4 condensate formation. We next expressed E2F4-GFP in Py8119 tumor cells, and consistent formation of condensates was observed (Fig. 3d). These condensates were markedly reduced by 5% 1,6-hexanediol (Hex hereafter),^45^ a compound presumed to disrupt weak hydrophobic interactions (Supplementary Fig. 6d). Notably, E2F4-Myc also formed condensates (Supplementary Fig. 6e), indicating that the puncta were not artificially formed by the GFP tag.

E2F4 contains several intrinsically disordered regions (IDRs) and domains critical for interactions with other proteins (Supplementary Fig. 6f, g). We aimed to identify which domain of E2F4 is essential for condensate formation and FAM114A1 binding. A series of E2F4 domain-deletion mutants was generated (Supplementary Fig. 6f) and transfected into Py8119 cells to assess their ability to form condensates. We did not observe significant differences in expression levels among these mutants (Supplementary Fig. 6h), whereas depletion of either IDRs (2–20 aa and 211–340 aa) or the dimerization domain (86–181 aa) significantly diminished E2F4 condensates (Fig. 3e and Supplementary Fig. 6i). Co-IP assays confirmed that the first IDR (2–20 aa) and the dimerization domain (86–181 aa) were critical for the interaction with FAM114A1 (Fig. 3f). These data suggest that FAM114A1 may bind and block E2F4 domains that are essential for its condensate formation, leading to fewer E2F4 puncta in cells. Next, we examined E2F4 condensates in samples from TNBC patients. Consistently, patients with high FAM114A1 expression presented significantly fewer E2F4 puncta (Fig. 3g, h). The specificity of E2F4 condensate staining was confirmed with E2F4-KD tumor cells and with isotype control IgG in patient samples (Supplementary Fig. 7).

**Fig. 4:**
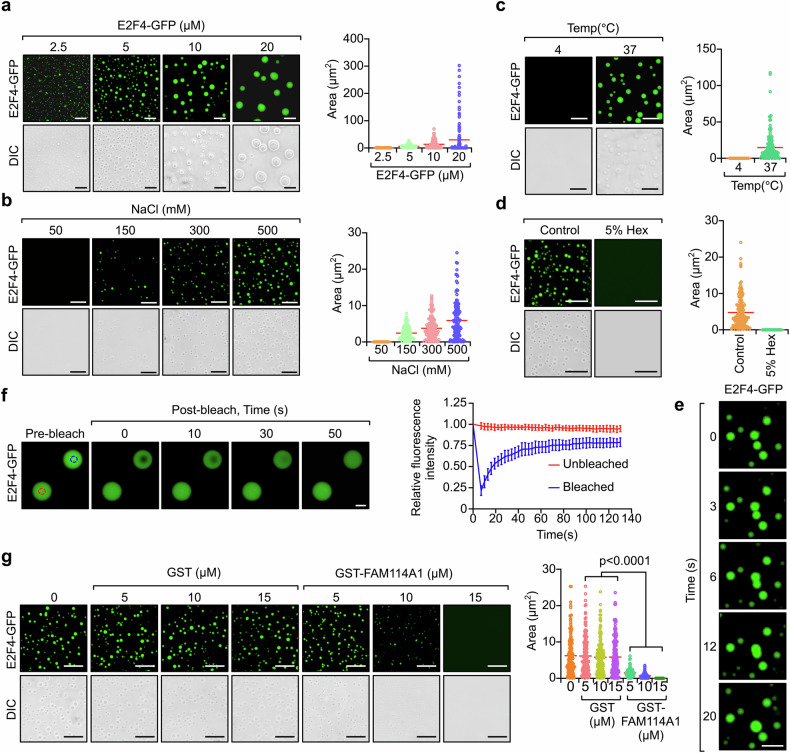
E2F4 condensates exhibit liquid-like properties and are suppressed by FAM114A1. **a** Droplet formation by E2F4-GFP was analyzed at the indicated concentrations with 500 mM NaCl at room temperature. E2F4-GFP (5 μM) was examined using droplet formation assays conducted at room temperature (**b**, **d**) or at 4 °C or 37 °C (**c**) with the indicated concentrations of NaCl (**b**) and with or without 5% Hex at 500 mM NaCl (**c**). For **a**–**d**, representative fluorescence and differential interference contrast (DIC) images of the droplets (left of each panel) and quantification of the size of the droplets (right of each panel) are shown. Each dot represents a droplet. Hex, 1,6-hexanediol; Bar, 20 µm. **e** Live-cell imaging of E2F4-GFP droplets. The arrows indicate representative fused E2F4 condensates. Bar, 10 µm. **f** E2F4-GFP droplets were subjected to a fluorescence recovery after photobleaching (FRAP) assay. The blue circle indicates the area for photobleaching, and the red circle indicates the area that served as a non-photobleaching control (left panel). The average values for the FRAP data are shown (right panel). Bar, 2 µm. **g** E2F4-GFP (5 μM) droplet formation was monitored in the absence or presence of the indicated concentrations of GST or GST-FAM114A1 recombinant proteins (left panel). The size of the droplets was quantified (right panel). Bar, 20 µm. The data represent the means and all the data points. The P-value was determined via one-way ANOVA (**g**)

In vitro assays were performed to further examine the properties of the E2F4 condensates. Purified E2F4-GFP (Supplementary Fig. 8a) spontaneously formed microsized droplets in solution, and the size and/or number of these droplets increased with increasing protein and salt concentrations (Fig. 4a, b). Moreover, higher temperatures promoted E2F4 droplet formation, and 5% Hex disrupted these droplets (Fig. 4c, d). These results suggest that hydrophobic interactions, rather than electrostatic interactions, mediate E2F4 droplet formation. In addition, we observed droplet fusion both in vitro and in cells (Fig. 4e and Supplementary Fig. 8b). Fluorescence recovery after photobleaching (FRAP) analysis was conducted on condensates formed in vitro and in intact cells. The rapid recovery of the fluorescence signal indicated highly dynamic diffusion of E2F4-GFP (Fig. 4f and Supplementary Fig. 8c, d). These data suggest that E2F4 condensates exhibit liquid-like properties. More importantly, consistent with the in vivo observations, the addition of the recombinant FAM114A1 protein significantly inhibited E2F4 droplet formation (Fig. 4g and Supplementary Fig. 8e). Taken together, these findings indicate that FAM114A1 binds to E2F4 and prevents its formation of a liquid-like condensate.

### E2F4 promotes MTDH expression and inhibits antigen presentation

**Fig. 5:**
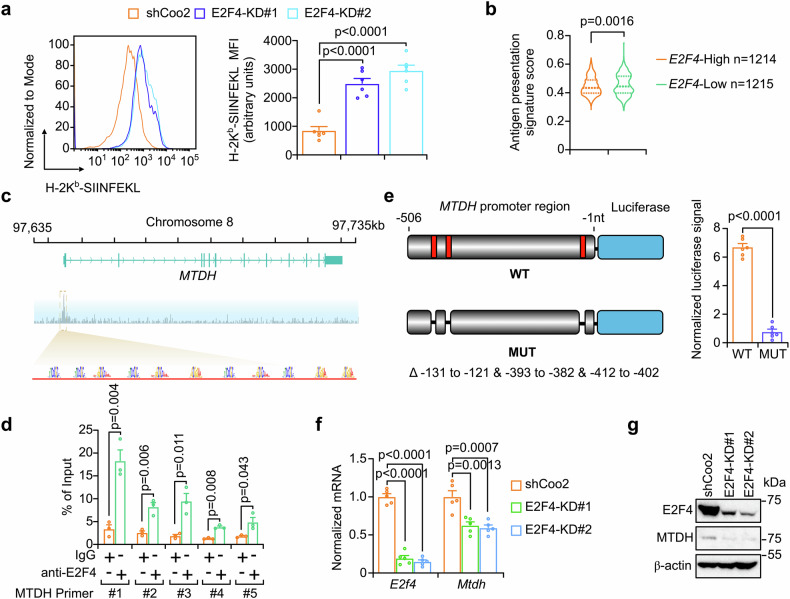
E2F4 transcriptionally drives MTDH expression to inhibit antigen presentation. **a** Py8119-OVA cells with (E2F4-KD#1 and KD#2) or without (shCoo2) stable E2F4 knockdown were cocultured with OT-I splenocytes for 2 h. Tumor cells were collected, and ovalbumin presentation was examined (left panel) and quantified (right panel) via flow cytometry. **b** scRNA-seq data of cancer cells extracted from TNBC patients. Cells without *E2F4* expression were excluded, and the remaining cells were stratified into high- and low-E2F4 expression groups on the basis of the median expression. Antigen presentation signature scores were calculated. **c** Browser tracks showing the promoter region of *MTDH*. E2F4 binding motif clusters were identified via MCAST with a motif p-value threshold of 0.005, a maximum gap threshold of 50 bp, and an E-value threshold of 10. **d** Chromatin immunoprecipitation (ChIP) assays were performed with an anti-E2F4 antibody or an IgG control. The samples were subjected to qPCR with the indicated primers that recognize the *MTDH* promoter region. **e** Schematic diagram of the firefly luciferase reporter with wild-type (WT) and mutant (MUT) *MTDH* promoter regions (−1 to −506 nt) (left panel). WT or MUT reporter plasmids were cotransfected with E2F4 and Renilla luciferase plasmids into Py8119 cells. Firefly luciferase activity was measured and normalized to Renilla luciferase activity (right panel). MUT, promoter regions of −131 to −121, −393 to −382, and −412 to −402 were depleted. Py8119-OVA cells with (E2F4-KD#1 and KD#2) or without (shCoo2) stable E2F4 knockdown were collected for RT‒qPCR or western blotting to detect E2F4 or MTDH at the mRNA (**f**) or protein level (**g**), respectively. The data represent the means ± SEMs. P-values were assessed using one-way ANOVA (**a**, **d**, **f**) or two-tailed Student’s *t*-test (**b**, **e**)

Next, we investigated whether such FAM114A1-suppressed E2F4 condensate formation contributes to immune evasion. Indeed, E2F4-KD enhanced tumor antigen presentation (Fig. 5a). In TNBC patients (NCT03197389), tumor cells with lower E2F4 expression presented a stronger antigen presentation signature (Fig. 5b), highlighting the possibility that E2F4 promotes immune evasion. To further explore this, we examined the direct transcriptional targets of E2F4. Chromatin immunoprecipitation (ChIP) sequencing and ChIP‒qPCR assays revealed that E2F4 binds to the promoter region of *MTDH* (Fig. 5c, d).^46^ The MCAST algorithm^47^ was employed to predict the motif clusters in the *MTDH* promoter region that bind to E2F4, resulting in the identification of 11 such clusters (Fig. 5c and Supplementary Fig. 9a). Luciferase reporter plasmids containing wild-type (WT) or mutant (MUT) *MTDH* promoter regions were constructed, and the reporter assays were performed (Fig. 5e and Supplementary Fig. 9a, b). A triple depletion mutant (MUT: deletion of nucleotides −131 to −121, −393 to −382, and −412 to −402) significantly attenuated the luciferase signal (Fig. 5e), suggesting that E2F4 binds to these regions of the *MTDH* promoter, thereby driving its transcription.

To confirm that E2F4 transcriptionally upregulates MTDH expression, the TNBC cell lines Py8119 and 4TO7 with and without E2F4-KD were collected to measure the mRNA and protein levels of MTDH. As expected, E2F4-KD significantly inhibited MTDH expression at both the mRNA and protein levels (Fig. 5f, g, and Supplementary Fig. 9c, d), and this inhibition was restored by reintroducing wild-type E2F4 (Supplementary Fig. 9e). Interestingly, none of the E2F4 domain-deletion mutants (Supplementary Fig. 6f) drove MTDH expression or restored E2F4-KD-induced MTDH downregulation (Supplementary Fig. 9e, f), suggesting that structural integrity may be required for *MTDH* transcriptional activation. Since MTDH has been reported to inhibit tumor antigen presentation,^26,48^ we speculated that E2F4-induced MTDH expression contributed to the suppression of antigen presentation.

### FAM114A1 activates E2F4-driven MTDH expression and immune suppression

**Fig. 6:**
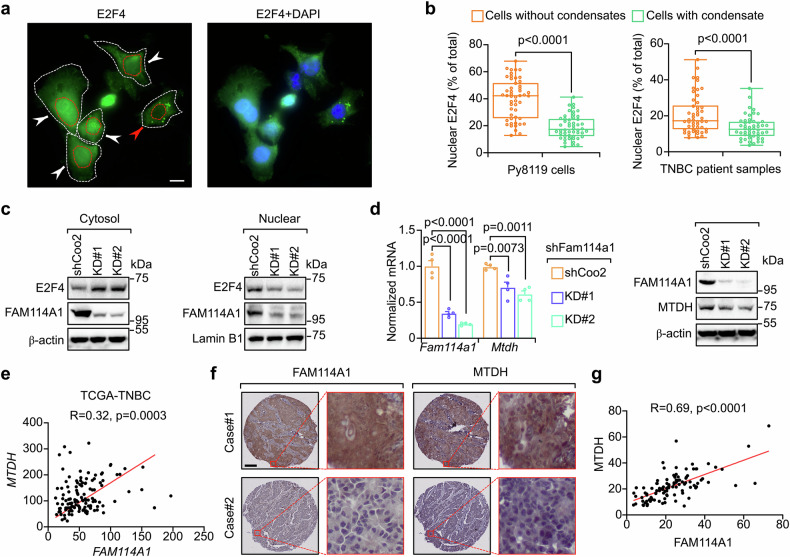
FAM114A1 enhances E2F4-mediated MTDH expression. **a** Py8119 cells were subjected to IF staining with an anti-E2F4 antibody. DNA was visualized with DAPI. The cells and nuclei are highlighted with white and red dashed circles, respectively. Bar, 10 µm. **b** The fluorescence intensity of nuclear E2F4 in Py8119 (left panel) and TNBC patient samples (right panel) was quantified. n = 50 cells in each group. **c**, **d** Py8119-OVA-Luc cells with (KD#1 and KD#2) or without (shCoo2) stable FAM114A1-knockdown were used to extract cytosolic or nuclear proteins. The expression of E2F4 and FAM114A1 in the cytosol and nucleus was examined via western blotting (**c**). The cells were subjected to RNA and protein extraction. The mRNA and protein levels of FAM114A1 and MTDH were examined via RT‒qPCR and western blotting, respectively (**d**). **e** RNA sequencing data of TNBC patients from the TCGA dataset were extracted. The Spearman rank correlation between *MTDH* and *FAM114A1* was plotted. n = 124 TNBC patients. **f**, **g** TNBC patient samples were employed for IHC staining with anti-FAM114A1 and anti-MTDH antibodies. Representative images are shown (**f**). The expression levels of FAM114A1 and MTDH were quantified with ImageJ and analyzed with Spearman’s rank correlation (**g**). n = 109 TNBC patients. Bar, 500 µm. The data represent the means ± SEMs. P-values were determined by two-tailed Student’s *t*-test (**b**) or one-way ANOVA (**d**)

Given that FAM114A1 binds to E2F4 and inhibits its liquid-like droplet formation, we wondered whether FAM114A1 was also involved in E2F4/MTDH signaling to suppress antigen presentation. Significantly lower levels of nucleus-localized E2F4 were observed in both mouse tumor cells and TNBC patient samples with condensates (Figs. 3g and 6a, b). E2F4-GFP mutants with varying droplet formation capabilities also confirmed that the ability of E2F4 to form liquid droplets in the cytoplasm may prevent its nuclear translocation (Supplementary Fig. 10a, b). Given that FAM114A1 prevents E2F4 liquid droplet formation, we examined whether FAM114A1 promotes E2F4 nuclear translocation. Tumor cells with FAM114A1-KD presented significantly lower E2F4 protein levels in nuclear fractions (Fig. 6c and Supplementary Fig. 10c), suggesting that FAM114A1 may facilitate E2F4 nuclear translocation. This is not surprising, as the E2F4 puncta formed upon FAM114A1-KD may be too large to pass through nuclear pores. In addition, E2F4 lacks a canonical nuclear localization signal, and its nuclear translocation depends on interactions with the transcription factor DP (TFDP) family and retinoblastoma (RB) family proteins (TFDP/RB proteins).^49^ Given that FAM114A1 binds to the domains at the N-terminal of E2F4 (Fig. 3f), particularly the dimerization domain (86–181 aa), which is also critical for its TFDP/RB interactions,^50^ we speculated that FAM114A1 may also facilitate binding between E2F4 and the TFDP/RB proteins, thereby increasing their nuclear translocation. To test this hypothesis, AlphaFold was employed to predict the binding affinity between the E2F4 and TFDP/RB proteins in the presence or absence of FAM114A1. As expected, the E2F4 and TFDP/RB proteins presented strong interactions, as evidenced by low ΔG and K_d_ values (Supplementary Table 4). Interestingly, the addition of FAM114A1 significantly decreased the ΔG and K_d_ values of the E2F4 and TFDP/RB protein complexes (Supplementary Table 4). Lower values of ΔG and K_d_ indicate stronger protein binding,^51^ suggesting that FAM114A1 facilitates interactions between the E2F4 and TFDP/RB proteins. However, this AlphaFold modeling predicted E2F4-TFDP/RB interactions require experimental validation, such as co-IP of endogenous E2F4-TFDP/RB complexes with/without FAM114A1. Nevertheless, these results reveal that FAM114A1 facilitates E2F4 nuclear localization by preventing its liquid droplet formation, and possibly also by enhancing its interaction with TFDP/RB proteins.

Since E2F4 transcriptionally activates MTDH, we hypothesized that FAM114A1-mediated E2F4 nuclear translocation enhances MTDH expression. In support of this notion, reduced MTDH expression was observed in tumor cells with FAM114A1-KD (Fig. 6d and Supplementary Fig. 10d). These observations were confirmed in TNBC patients, as the expression levels of FAM114A1 and MTDH were positively correlated (Fig. 6e–g and Supplementary Table 1). To confirm that MTDH is involved in FAM114A1- and/or E2F4-mediated antigen presentation signaling, tumor cells with FAM114A1 or E2F4-KD were rescued with ectopic MTDH expression. The increase in antigen presentation induced by FAM114A1 or E2F4-KD was partially reversed by ectopic MTDH expression (Supplementary Fig. 10e, f), suggesting that FAM114A1 and E2F4 partially exert their immunosuppressive effects via MTDH. Taken together, in addition to activating the PI3K/AKT pathway, FAM114A1 also inhibits antigen presentation by promoting E2F4-driven MTDH expression.

### FAM114A1-mediated immunosuppression is independent of its cell cycle regulation

**Fig. 7:**
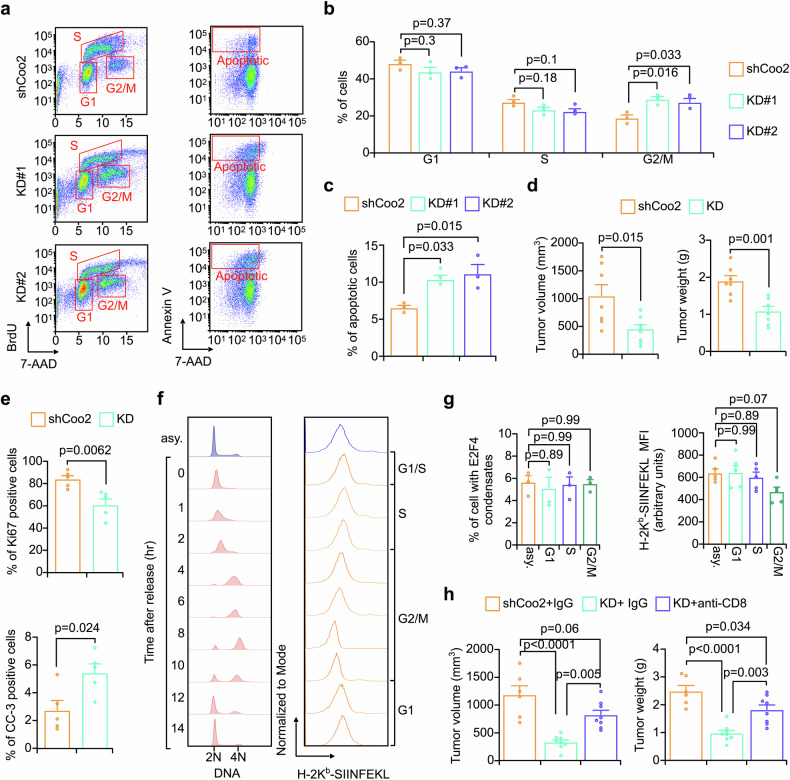
FAM114A1-mediated immunosuppression is independent of its ability to regulate the cell cycle. **a‒c** Py8119-OVA-Luc tumor cells with FAM114A1-knockdown and the corresponding controls were used for the tumor sphere assay. Five days after culture, the spheres were collected for cell cycle and apoptotic analysis (**a**), and the proportion of cells in each phase and the proportion of apoptotic cells were quantified (**b**, **c**). n = 3 replicates per group. shCoo2: Py8119-OVA-Luc PLKO-shCoo2; KD#1: Py8119-OVA-Luc PLKO-shFAM114A1#1; KD#2: Py8119-OVA-Luc PLKO-shFAM114A1#2. **d**, **e** Py8119 tumor cells with and without FAM114A1-knockdown were orthotopically injected into nude mice. Tumor size and weight were evaluated at the endpoint (**d**). n = 7 and 8 mice for the control (shCoo2) and FAM114A1-knockdown (KD) groups, respectively. Tumors from the indicated groups were collected for IHC staining with anti-Ki67 and cleaved caspase 3 (CC-3) antibodies. The percentage of positive cells was determined (**d**). n = 5 tumors per group. **f** Py8119 cells were synchronized with a double thymidine block. The cells released at the indicated time points were subjected to flow cytometry analysis to examine the cell cycle distribution and antigen presentation status. asy. : asynchronized. **g** Cells at the indicated phases were subjected to immunofluorescence staining with an anti-E2F4 antibody. The percentage of cells with E2F4 condensates at the indicated phases was quantified (left panel). The antigen presentation status of cells at the indicated phases was quantified (right panel). **h** OT-I female mice were pretreated with 125 μg/mouse anti-CD8 antibody or the corresponding isotype control every two days for one week. Py8119-OVA-Luc tumor cells with/without FAM114A1-knockdown were orthotopically injected into the mice, and the mice were continually treated with 125 μg/mouse of anti-CD8 antibody or the corresponding isotype control twice per week until the end of the experiment. The tumor size was measured 6 weeks after tumor cell injection. Primary tumors were dissected and weighed at week 7. n = 6 mice for the FAM114A1 control (shCoo2)- and isotype (IgG)-treated groups; n = 8 mice for the FAM114A1-knockdown (KD)- and isotype (IgG)- or anti-CD8 antibody-treated groups. The data represent the means ± SEMs. P-values were determined via one-way ANOVA (**b**, **c**, **g**, **h**) or two-tailed Student’s *t*-test (**d**, **e**)

Both PI3K/AKT and E2F4 have been reported to be critical for cell cycle regulation.^50,52^ Considering that FAM114A1 targets these two pathways, we examined whether FAM114A1 is also involved in cell cycle progression. Pathway enrichment analysis revealed that cell cycle-related signaling was enriched in FAM114A1-KD/low tumors (Supplementary Fig. 4a, c) and in potential FAM114A1-binding partners (Supplementary Fig. 5e), suggesting that FAM114A1 might regulate cell cycle progression. To experimentally confirm this, we performed in vitro tumor sphere assays in the absence of immune cells. FAM114A1-KD significantly decreased sphere numbers and sizes, suggesting that FAM114A1 impacts cell proliferation and/or cell viability (Supplementary Fig. 11a, b). Cell cycle and apoptosis analyses indicated that FAM114A1-KD resulted in more cells in the G2/M phase and increased apoptosis (Fig. 7a–c). In vivo experiments in immunodeficient mice further supported that FAM114A1-KD suppressed cell proliferation and increased apoptosis (Fig.7d, e, and Supplementary Fig. 11c). It is not surprising that FAM114A1 impacts cell cycle progression and cell viability. Both PI3K/AKT^52^ and MTDH^53^ are critical for cell cycle progression, and targeting these proteins results in G2/M phase arrest, as described in previous studies. Since FAM114A1-KD inhibits PI3K/ATK signaling (Fig. 2) and downregulates MTDH (Fig. 6), we expected similar effects.

We next asked whether such FAM114A1-mediated cell cycle regulation also contributed to E2F4 condensate suppression and antigen presentation inhibition. To address this, Py8119 cells were synchronized with a double thymidine block, and the E2F4 condensate formation status at different cell cycle phases was examined (Fig. 7f, left panel). Puncta can be observed at each phase at similar ratios (Fig. 7g, left panel and Supplementary Fig. 12a). These findings suggest that E2F4 has constant condensate formation ability during cell cycle progression and that FAM114A1-KD-induced cell cycle defects do not directly contribute to increased E2F4 condensates. Consistent with this notion, the recombinant FAM114A1 protein significantly attenuated E2F4 liquid-like condensate formation in a cell-free system (Fig. 4g), suggesting that FAM114A1 directly suppresses E2F4 condensate formation.

Interestingly, antigen presentation fluctuated during cell cycle progression, with the G2/M phase showing relatively lower antigen presentation status (Fig. 7f, right panel and Fig. 7g, right panel). The antigen presentation defects caused by cell cycle inhibition have been reported in previous studies.^54^ Notably, FAM114A1-KD resulted in more cells in the G2/M phase (which corresponds to less antigen presentation) (Fig. 7a, b); however, greater antigen presentation was observed in these cells (Supplementary Fig. 5a), suggesting that the increased antigen presentation upon FAM114A1-KD is not mediated by its cell cycle regulation. In addition, we examined MTDH expression during cell cycle progression, and no significant changes were detected (Supplementary Fig. 12b). These results indicate that the lower MTDH protein levels and the consequent elevation in antigen presentation upon FAM114A1-KD are not directly caused by cell cycle defects.

To distinguish the effects of FAM114A1 on cell cycle progression from its immunosuppressive role, we developed an in vitro tumor sphere/immune coculture assay. After the spheres were established, OT-I splenocytes were added, and coculture was conducted. The remaining viable tumor cells with/without coculture, and with/without FAM114A1-KD, were assessed by a luciferase assay at the endpoint. Compared with the corresponding control, the FAM114A1-KD groups presented fewer viable cells under both coculture and non-coculture conditions, with the coculture group exhibiting a significantly greater difference (Supplementary Fig. 13a, left panel). Moreover, after normalization to the corresponding non-coculture group to eliminate the tumor intrinsic effects of FAM114A1, the FAM114A1-KD group still presented fewer viable tumor cells upon immune cell challenge (Supplementary Fig. 13a, right panel), suggesting that, in addition to its intrinsic role in tumors, FAM114A1-mediated immune suppression plays a pivotal role in breast cancer progression. To confirm this conclusion in vivo, OT-I mice were pretreated with either an anti-CD8 antibody or isotype IgG to deplete CD8^+^ T cells or served as controls. The mice were then inoculated with FAM114A1-KD or control tumor cells and continuously treated with IgG or the anti-CD8 antibody. CD8^+^ T-cell depletion significantly restored tumor progression in the FAM114A1-KD group (Fig. 7h and Supplementary Fig. 13b), suggesting that FAM114A1-mediated immune suppression is critical for its tumor-promoting function. In summary, although FAM114A1 is involved in cell cycle regulation, its antigen presentation inhibition and consequent immunosuppressive effects are independent of its intrinsic effects on tumor cells.

### FAM114A1-targeting sensitizes TNBC to ICB therapy

To understand whether FAM114A1-mediated immune evasion depends on its ability to suppress antigen presentation, Py8119-OVA-Luc cells with β2-microglobulin (B2m) and FAM114A1-knockdown, either alone or in combination, were generated (Supplementary Fig. 14a). The cells were subjected to a coculture assay and flow cytometry analysis to assess antigen presentation. As a key component of the MHC-I complex,^55^ B2m-KD significantly disrupted antigen presentation and reduced consequent immune killing (Supplementary Fig. 14b, c). On the basis of B2m-KD, FAM114A1-KD did not further enhance the immune killing of tumor cells (Supplementary Fig. 14b, c). These findings suggest that FAM114A1 inhibits immune killing by suppressing tumor antigen presentation.

**Fig. 8:**
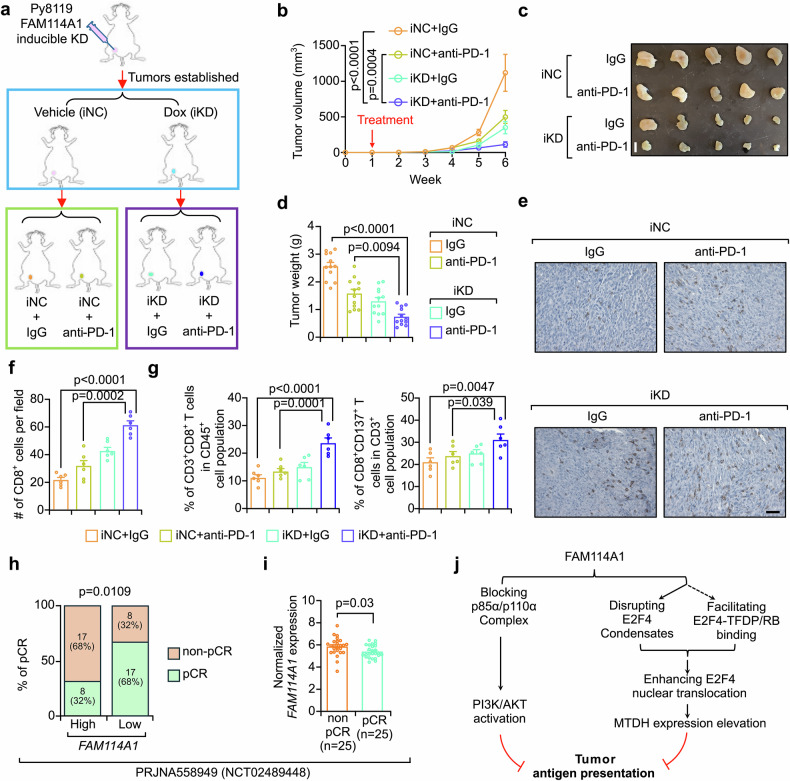
FAM114A1 targeting sensitizes TNBC to ICB therapy. **a**, Schematic diagram of FAM114A1-inducible knockdown (iKD) combined with anti-PD-1 therapy in mouse models. **b**–**d** Py8119-OVA-Luc cells with inducible FAM114A1-knockdown were generated. The cells were orthotopically injected into the mammary fat pads of female OT-I mice. One week after the injection (when the tumors were established), the mice were administered doxycycline (iKD) and anti-PD-1 antibodies alone or in combination. Control mice received either an isotype control antibody (IgG) and/or vehicle (iNC). Tumor growth was monitored and measured weekly (**b**). The tumors were dissected at week 7, and representative tumors are shown (**c**). Tumor weight was measured (**d**). n = 12 mice per group. Bar, 1 cm. **e**–**g** Tumors from (**b**) were collected for IHC staining with an anti-CD8 antibody (**e**). The percentage of CD8^+^ T cells per field was quantified in each group (**f**). Tumors were digested, and flow cytometry assays were performed to examine the ratio of CD8^+^ T cells (CD3^+^CD8^+^) to activated CD8^+^ T cells (CD8^+^CD137^+^) in tumors (**g**). n = 6 tumors per group. Bar, 50 µm. **h**, **i** *FAM114A1* expression was examined in the NCT02489448 immunotherapy TNBC patient cohort. The median expression level was used as the cutoff to define high- and low-FAM114A1 patients. The percentages of pathological complete response (pCR) and non-pCR patients are shown for both *FAM114A1*-high and -low patients (**h**). The expression of *FAM114A1* in pCR patients and non-pCR patients is shown (**i**). **j** Schematic diagram of the working model. The data represent the means ± SEMs. P-values were determined via one-way ANOVA (**b, d, f, g**), the χ^2^ test (**h**), and two-tailed Student’s *t*-test (**i**)

To evaluate the therapeutic potential of targeting FAM114A1, antisense oligonucleotides (ASOs) that could effectively KD endogenous FAM114A1 were designed and synthesized (Supplementary Fig. 14d). Treatment with these FAM114A1 ASOs significantly enhanced the tumor immune killing effect in our coculture system (Supplementary Fig. 14e). Next, we investigated whether targeting FAM114A1 could enhance ICB therapy in TNBC. To address this, Py8119 cells were used to generate FAM114A1-inducible KD cell lines, in which endogenous FAM114A1 can be effectively knocked down upon doxycycline (Dox) treatment (Supplementary Fig. 14f). As expected, induced FAM114A1-KD also sensitized tumor cells to immune killing (Supplementary Fig. 14g). We next performed in vivo experiments in mouse models. After tumors became palpable, mice orthotopically inoculated with Py8119 cells harboring FAM114A1-inducible KD were subjected to Dox and anti-PD-1 treatment, either individually or in combination. Isotype IgG and vehicle for Dox served as controls in this setting (Fig. 8a). Inducing FAM114A1-KD together with anti-PD-1 treatment significantly inhibited tumor progression (Fig. 8b–d). Consistently, tumors from the FAM114A1-KD and anti-PD-1 combination treatment group presented significantly higher CD8^+^ T-cell infiltration and activation than did those from the anti-PD-1 treatment alone group (Fig. 8e–g). Overall, targeting FAM114A1 in tumor cells sensitizes TNBC to ICB therapy.

To validate these findings in TNBC patients, data from four cohorts (PRJNA558949,^56^ GSE173839,^57^ GSE194040,^58^ and GSE169246^24^) with response information from ICB treatment were extracted for subsequent analysis (Supplementary Table 1). Patients with high *FAM114A1* expression in multiple cohorts presented lower pathological complete response (pCR) rates upon immunotherapy (Fig. 8h and Supplementary Fig. 14h, left panels). Consistently, patients who achieved pCR had lower *FAM114A1* expression (Fig. 8i and Supplementary Fig. 14h, right panels). Moreover, the FUSCC dataset (SRP157974) was used again to compare further characteristics, such as tumor stage, *PDCD1*, and *CD274*, in the *FAM114A1*-high/low patient groups. Tumor stage and *CD274* expression were not correlated with the *FAM114A1* level (Supplementary Table 5). Interestingly, patients with low-*FAM114A1* expression presented higher *PDCD1* expression after correction for other confounders (Supplementary Table 5), supporting our finding that tumors with lower FAM114A1 expression had a better response rate to anti-PD-1 therapy (Fig. 8b–i). Furthermore, *FAM114A1*-low patients presented significantly greater CD8⁺ T-cell infiltration (Supplementary Table 5), independent of tumor stage, and *CD274* and *PDCD1* expression, according to multivariate logistic regression analysis. Overall, FAM114A1 may be an independent factor that predicts ICB treatment response rates in TNBC patients.

To examine and improve the ability of FAM114A1 to predict the ICB response, we sought to develop a FAM114A1 signature. To this end, tumor cells from scFFPE-Seq data were extracted to identify differentially expressed genes upon FAM114A1-KD. Genes downregulated in the FAM114A1-KD group and together with genes in the FAM114A1 downstream signaling axes, such as PI3K/AKT, E2F4, and MTDH, were selected to develop an ICB response scoring model (see details in the “Methods” section). The selected genes were constructed into a FAM114A1-centric molecular signature (Supplementary Table 6), and on the basis of our mechanistic studies above, a lower score suggests a better ICB response. As expected, the FAM114A1 signature indeed showed strong predictive performance for ICB treatment efficacy in TNBC patients across multiple cohorts (Supplementary Fig. 14i). Its predictive performance is superior to that of PD-1 or PD-L1 alone, which are currently used as predictive biomarkers for the efficacy of ICB in the clinic,^59,60^ suggesting that the FAM114A1 signature could help precisely identify TNBC patients who may benefit from ICB treatment.

## Discussion

ICB therapy is a revolutionary treatment strategy that has been applied to many cancer types,^61,62^ including TNBC.^63^ Unfortunately, the vast majority of TNBC patients are either ineligible for this therapy or exhibit a limited response rate.^64^ The precise identification of patients who may benefit from this treatment and strategies to overcome therapeutic resistance are urgently needed. In this study, we constructed a FAM114A1 signature capable of identifying TNBC patients who may benefit from ICB therapy. Upon ICB treatment, many patients develop diverse mechanisms that lead to resistance.^65^ Accumulating evidence has demonstrated that biomolecules aggregate into condensates to promote pathological processes, including resistance to cancer immunotherapy.^11,13,14^ Here, we found that E2F4 assembled into liquid-like droplets in the cytoplasm, which impaired its nuclear translocation and subsequent MTDH expression and suppressed antigen presentation (Figs. 5 and 6). FAM114A1 prevented the assembly of such liquid-like droplets to increase E2F4-driven MTDH expression, thereby inhibiting antigen presentation and leading to resistance to ICB treatment (Figs. 3–8). Our study explored liquid-like condensate formation dynamics and offered new perspectives for understanding antitumor immunity (Fig. 8j and Supplementary Fig. 15).

Previous studies have indicated that FAM114A1 is involved in cardiac pathological remodeling and promotes the development of cardiovascular disease.^66^ In addition, preliminary correlation studies suggest that high expression of FAM114A1 is associated with a poor prognosis in cancers,^67,68^ and that AKT1 signaling might be involved in the tumor-promoting role of FAM114A1 in hepatocellular carcinoma.^69^ However, the precise molecular mechanisms governing the oncogenic functions of FAM114A1 remain poorly understood. Here, we found that FAM114A1 is involved in cell cycle regulation and antitumor immunity. Many pathways (Supplementary Fig. 4) and cell populations (such as fibroblasts) (Fig. 1g and Supplementary Fig. 3a) are involved in the tumor-promoting role of FAM114A1, suggesting that FAM114A1 is a pleiotropic protein. Notably, ASO-based in vitro tests and doxycycline-inducible KD-based in vivo validation demonstrated the therapeutic potential of targeting FAM114A1 to sensitize TNBC patients to ICB.

P85α is a repressive regulatory subunit that binds to and inhibits the catalytic activity of the p110α subunit, consequently resulting in reduced PI3K activation.^70^ Here, we found that FAM114A1 competes with p110α to bind p85α, and that the expression or addition of FAM114A1 blocks the p85α/p110α interaction (Fig. 2g, h), which could be the mechanism underlying FAM114A1-mediated PI3K/AKT activation. Another study indicated that monomeric free p85α is a negative regulator of PI3K signaling.^71^ The binding of FAM114A1 to p85α may also result in a reduction in monomeric free p85α, thereby enhancing PI3K/AKT activation. Notably, both p85 and p110 have multiple family members, such as p85α, p85β, p110α, p110β, and p110δ,^72,73^ which can interact with each other through conserved domains. We did not examine whether FAM114A1 blocks the binding of p85α to other p110 subunits. Given that FAM114A1 binds to the nSH2, iSH2, and cSH2 domains of p85α (Fig. 2f), we speculate that FAM114A1 may also disrupt the interactions between p85α and other p110 subunits, as these domains are critical for its binding to all forms of p110 subunits.^74,75^ Moreover, FAM114A1 may also bind to other p85 subunits or even p55 regulatory subunits, as they all contain conserved nSH2, iSH2, and cSH2 domains.^76^ As these repressive regulatory subunits bind to and inhibit catalytic subunits in general, FAM114A1 can activate the PI3K pathway by blocking such interactions between regulatory and catalytic subunits.

PI3K/AKT activation is involved in cancer progression; unfortunately, TNBC patients show limited response rates to the PI3K/AKT-targeting treatments,^25^ suggesting that other pathways may compensate for PI3K/AKT inhibition. In line with this notion, our results indicate that tumor cells utilize both the PI3K/AKT pathway and the E2F4-driven MTDH pathway to escape immunosurveillance. Targeting PI3K/AKT may not be sufficient, as tumor cells can exploit E2F4/MTDH to develop resistance. E2F4 was initially identified as a transcriptional repressor;^77^ however, accumulating evidence suggests that E2F4 also promotes gene transcription.^50,78–80^ Moreover, E2F4 was found to promote cancer progression,^81^ and more importantly, the expression of E2F4 was negatively correlated with CD8^+^ T-cell infiltration in head and neck squamous cell carcinoma and hepatocellular carcinoma.^82,83^ Consistent with these findings, we demonstrated that E2F4 transcriptionally activated MTDH, thereby increasing its expression (Fig. 5 and Supplementary Fig. 9). E2F4 binding at the promoter region of MTDH was also reported in a study in which E2F4 targets were screened via ChIP-Seq at the genome scale.^84^ These findings support our data that E2F4 binds to the MTDH promoter and enhances MTDH expression. In addition, the consequent suppression of antigen presentation, mediated by elevated MTDH expression,^26^ may also explain the negative correlation between E2F4 and immune cell infiltration observed in other studies. Previous studies have indicated that E2F4 forms aggregates in the cytoplasm, which are important for centriole amplification.^85^ Here, we demonstrated that E2F4 formed condensates in the cytoplasm (Fig. 3 and Supplementary Fig. 6), and these condensates presented liquid-like properties (Fig. 4 and Supplementary Fig. 8). More importantly, we observed a negative correlation between E2F4 droplet formation and E2F4 nuclear localization (Fig. 6 and Supplementary Fig. 10). We speculate that the condensates of E2F4 structurally attenuate its nuclear translocation. FAM114A1 prevents its droplet formation (Figs. 3 and 4) and therefore results in increased nuclear E2F4 levels (Figs. 5 and 6). Notably, E2F4 does not have a nuclear localization signal peptide, and its nuclear translocation depends on its interaction with the TFDP/RB proteins.^50,86^ Given that the dimerization domain of E2F4 is critical for its interactions with both FAM114A1 (Fig. 3 and Supplementary Fig. 6) and TFDP/RB proteins,^50^ FAM114A1 may facilitate binding between E2F4 and the TFDP/RB proteins to promote their nuclear translocation. Consistent with this notion, FAM114A1 indeed increased the interaction between E2F4 and the TFDP/RB proteins according to our AlphaFold modeling predictions (Supplementary Table 4).

In conclusion, we demonstrate that FAM114A1 promotes TNBC immune evasion by suppressing tumor antigen presentation. Mechanistically, FAM114A1 activates the PI3K/AKT pathway by interacting with p85α, and concurrently increases E2F4 nuclear localization, and consequently, MTDH expression. These two mechanisms converge to regulate cell cycle progression, and more importantly, inhibit tumor antigen presentation (Fig. 8j and Supplementary Fig. 15). However, further studies are needed to investigate whether these two mechanisms are functionally independent or synergistic. Building upon these findings, we developed a FAM114A1 signature that can identify TNBC patients who may respond to ICB therapy. Our study elucidates a dual-pathway mechanism through which FAM114A1 orchestrates immune evasion in TNBC by impairing antigen presentation and establishes a clinically implementable signature for precision immunotherapy stratification in TNBC patients.

## Materials and methods

### Cell lines

HEK293T, MDA-MB-231, and 4TO7 cells were cultured in DMEM supplemented with 10% FBS, 2 mM glutamine, and 100 U penicillin/0.1 mg/ml streptomycin. H29 cells expressing retroviral packaging components were maintained in DMEM containing 10% FBS, 2 mM glutamine, 100 U penicillin/0.1 mg/ml streptomycin, 1 µg/ml doxycycline, 2 µg/ml puromycin, and 300 µg/ml G418. Py8119 cells were maintained in DMEM/F12 (1:1) medium supplemented with 10% fetal bovine serum (FBS), 20 ng/mL epidermal growth factor (EGF), 5 µg/mL insulin, 2 µg/mL hydrocortisone, 100 U/mL penicillin, and 0.1 mg/mL streptomycin. All cell lines were routinely tested for mycoplasma contamination and authenticated. Mouse splenocytes freshly isolated from OT‑I mice were cultured in RPMI‑1640 medium containing 10% FBS, 1% HEPES, 1% sodium pyruvate, 50 µM β‑mercaptoethanol, 100 U/mL penicillin, and 0.1 mg/mL streptomycin.

### Animal models

All animal experiments were approved by the Institutional Animal Care and Use Committee (IACUC) and carried out in accordance with the institutional guidelines of Fudan University Shanghai Cancer Center and Princeton University. BALB/c-Nude (strain no. D000521) mice were obtained from GemPharmatech. C57BL/6 OT-I mice were obtained from Jackson Laboratory (Stock No: 003831), and C57BL/6 albino (Cat. NO. NM-KO-225156) mice were purchased from Shanghai Model Organisms Center. We crossed the two strains to obtain albino;OT-I mice (termed OT-I), in which the pigment is completely absent from the hair, and convenient for cell injection and tumor monitoring. For CD8⁺ T‑cell depletion, mice received 125 µg per mouse of anti‑CD8⁺ T‑cell depletion antibody (Bio X Cell, #BE0061) or an equal amount of isotype control antibody (Bio X Cell, #BE0090) in PBS via intraperitoneal injection three days prior to tumor cell inoculation. Antibody administration was continued twice per week following tumor cell injection. For spontaneous tumorigenesis studies, 4–6‑week‑old female nude mice or OT‑I mice were anesthetized, and a small incision was made to expose the mammary gland. A 10 µL single‑cell suspension in PBS:Matrigel (1:1) was injected into the inguinal (#4) mammary fat pad. Mice were examined weekly for mammary tumor development. Tumors were considered established after being palpable for two consecutive weeks. Tumor size was measured with calipers, and volume was calculated using the formula: (length × (width²) / 2).

### Single-cell gene expression Flex assay

Primary tumors from control (shCoo2), FAM114A1-KD#1, and FAM114A1-KD#2 Py8119 cell-inoculated mice were collected and processed into formalin-fixed paraffin-embedded (FFPE) tumors. Six control FFPE tumors were combined into one group termed the shCoo2 group. Three FAM114A1-KD#1 FFPE tumors and three FAM114A1-KD#2 FFPE tumors were combined into the FAM114A1-KD group. shCoo2 and FAM114A1-KD FFPE samples were thawed and divided equally for processing via standard 10X Flex assays, following the recommended protocols. Single-cell RNA sequencing was performed with 10X Chromium Single-cell Gene Expression Flex on the Illumina NovaSeq 6000 platform (Berry Genomics Corporation, Beijing, China). The raw reads were aligned with CellRanger (v9.0.1, GRCm39 reference), and the count matrices were processed in Seurat (v5.0.0.1, R v4.3.3).^87^ Low-quality cells (<200 or >6000 UMIs, >10% mitochondrial genes) and doublets (DoubletFinder v2.0.3)^88^ were excluded. The data were normalized via Seurat’s NormalizeData.

For single-cell analysis, 2000 variable genes were identified, PCA was applied (regressing for UMIs), and the top 30 PCs were used for clustering and UMAP. The cell types were annotated via marker genes, and the DEGs were identified via Seurat’s FindAllMarkers. T cells were clustered via Seurat’s elbow plot and annotated (CD4^+^ T: *Cd4*, CD8^+^ T: *Cd8a*, NKT: *Ncr1/Klrb1c*, γδT: *Trdc*). CD8^+^ T cells were further subclustered into activated (high *GZMB/IFNG*) and nonactivated subsets. The activation status of shCoo2- and FAM114A1-KD CD8^+^ T cells was compared via genes from the “GSE15324_NAIVE_VS_ACTIVATED_CD8_TCELL_UP” gene set from MSigDB (v2023.2)^89^ and visualized via a heatmap (pheatmap v1.0.12).

### Calculation of signature scores

GSVA^90^ was employed to calculate signature enrichment scores on the basis of single-cell sequencing data. The analysis focused on cancer cells from both flex single-cell data and single-cell data from NCT03197389 clinical samples,^34^ using gene sets for antigen presentation (“GOBP_ANTIGEN_PROCESSING_AND_PRESENTATION”) and the PI3K/AKT pathway (“GOBP_POSITIVE_REGULATION_OF_PHOSPHATIDYLINOSITOL_3_KINASE_ACTIVITY”) from MSigDB. Additionally, TNBC patients from the TCGA BRCA dataset (UCSC Xena platform)^91^ were analyzed via the “GSE15324_NAIVE_VS_ACTIVATED_CD8_TCELL_UP” gene set (MSigDB v2023.2) to evaluate pathway activity in activated CD8^+^ T cells via GSVA.

### ICB therapy efficacy prediction and ROC analysis

Three TNBC immunotherapy patient cohorts (PRJNA558949, ISPY2 2021 (GSE173839), and 2022 (GSE194040))^56–58^ with bulk RNA sequencing data or microarray data, were employed. We employed a random forest algorithm to predict the ICB response, with the following key steps to ensure model robustness: **Data Sources**: This study analyzed gene expression data from three independent cohorts: 21 TNBC patient samples from ISPY2 2021 (GSE173839), 29 TNBC patient samples from ISPY2 2022 (GSE194040), and 50 TNBC patient samples from the NCT0240488 (PRJNA558949) clinical trial. **Feature Selection and Data Splitting**: In each iteration, key genes such as FAM114A1 were always included, and additional candidate genes were randomly selected as features. The combined 100 samples were then randomly divided into two independent subsets (df1 and df2), each further split into training and testing sets at a 40%:60% ratio, ensuring independence between training and testing samples. **Validation Strategy and Overfitting Prevention**: Regarding cross-validation, we implemented a repeated random sampling approach. Specifically, the entire modeling process was repeated 1000 times with different random seeds for feature selection and data partitioning, generating 1000 different train‒test combinations. This approach offers several advantages over traditional k-fold cross-validation: (i) it provides a more comprehensive assessment of model performance variability; (ii) it enhances result reliability through dual test set validation; and (iii) it avoids dependence on specific data splitting schemes. By analyzing the distributions of the AUC, accuracy, and kappa statistics across 1000 iterations, we can assess model stability and effectively prevent overfitting. **Model training and evaluation**: In each iteration, a random forest model (ntree=500) was trained on the training set and evaluated on two independent test sets. The performance metrics included the AUC, accuracy, and kappa statistic, providing a comprehensive assessment of the model’s discriminative power and consistency. **Model Stability**: We further validated model stability by repeating the training and evaluation process with 1000 different random seeds and summarizing the distribution of performance metrics.

### Immunohistochemistry (IHC) staining and classification

Formalin-fixed paraffin-embedded (FFPE) TNBC patient sections were obtained from Fudan University Shanghai Cancer Center (FUSCC), with a total of 109 patients (supplementary Table 2). Our study was approved by the independent ethics committee/institutional review board of FUSCC (Shanghai Cancer Center Ethics Committee). All patients provided written informed consent before inclusion. For mouse samples, tumors were collected from each indicated experiment and processed into FFPE sections. Formalin‑fixed, paraffin‑embedded (FFPE) tumor tissue sections (4 µm thickness) were subjected to IHC staining to evaluate the expression of FAM114A1, CD8, phospho‑AKT (Ser473), and MTDH. Staining was performed using a Ventana BenchMark ULTRA automated immunostainer (Ventana Medical Systems, Tucson, AZ, USA). The following primary antibodies were used: FAM114A1: HPA069701 (Sigma; 1:200 dilution for human specimens) and 67926‑1‑Ig (Proteintech; 1:200 dilution for mouse specimens). CD8: SP57 (Ventana; undiluted for human specimens) and 98941S (Cell Signaling Technology, CST; 1:100 dilution for mouse specimens). Phospho‑AKT (Ser473): 4046 (CST; 1:200 dilution). MTDH: AMAB90762 (Sigma; 1:500 dilution). Sections were incubated with biotinylated goat anti‑rabbit IgG (H + L) secondary antibody (Vector Laboratories, BA‑1000; 1:300 dilution). Images were acquired using Carl Zeiss Zen software (version 3.0) and processed using ImageJ (bundled with Java 1.8.0_172).

IHC analysis revealed that FAM114A1, phospho‑AKT (Ser473), and MTDH expression were predominantly localized to tumor cells. Protein levels were quantified using the ImageJ color deconvolution tool. CD8 positivity was primarily observed in tumor‑infiltrating lymphocytes (TILs), and the CD8 expression level was calculated as the percentage of CD8‑positive cells (number of positive cells/total cells). A cutoff value of ≥5% was used to classify samples as CD8⁺ high. All stained sections were independently evaluated by two experienced pathologists blinded to the patients’ clinical data. Discrepancies in scoring were resolved through joint review and consensus.

### Immunofluorescence (IF) staining and IF-based classification

The same set of patient FFPE sections was used for IF staining. Cells with or without E2F4-GFP or E2F4-Myc plasmid transfection were fixed with 4% formaldehyde, blocked with 5% BSA, and subjected to IF staining. For cells not transfected or transfected with E2F4-Myc, anti-E2F4 (10923-1-AP, Proteintech, 1:20 dilution), anti-IgG (98136-1-RR, Proteintech, 1:50 dilution), and anti-Myc (16286-1-AP, Proteintech, 1:100 dilution) antibodies were used for staining. The primary antibody-incubated samples were incubated with goat anti-rabbit IgG H&L (FITC) (MCE, HY-P80951, 1:100 dilution). All the samples were stained with 300 nM DAPI to visualize the DNA. Images were taken with a Leica Stellaris 5 microscope and processed with Leica Application Suite X (LAS X). E2F4 condensates in each cell were counted. Cells with ≥10 condensates were defined as condensate-positive cells. For FFPE samples, IgG isotype staining was performed to exclude nonspecific staining, and only the cells with ≥10 condensates were determined to be E2F4 condensate-positive cells.

### Plasmid construction, viral production, and infection

pLKO plasmids encoding shRNAs targeting murine *FAM114A1* (FAM114A1-KD#1, TRCN0000174803; and FAM114A1-KD#2, TRCN0000174459), murine *E2f4* (E2F4-KD#1, TRCN0000348953; and E2F4-KD#2, TRCN0000331779), and murine *B2m* (B2m-KD#1, TRCN0000288438; and B2m-KD#2, TRCN0000295705) were purchased from Genewiz (Suzhou, China) and were cloned as described previously.^92,93^ For FAM114A1-inducible knockdown cell lines, the same two targets were used and cloned and inserted into the pTRIPZ vector (Addgene#127696). For FAM114A1 CRISPRa guide RNA cloning, the three guide RNAs that target FAM114A1 (supplementary Fig. 1e) were cloned and inserted into the lenti sgRNA cloning backbone (Addgene#73797) as previously described.^27^ For the overexpression and rescue experiments, genes encoding FAM114A1, MTDH, E2F4, p85α, and p110α were cloned from HEK293T cDNA. E2F4-GFP wild-type or mutant forms were cloned and inserted into the pEGFP-N1 vector, and the remaining genes (wild-type or mutant forms) were cloned and inserted into the pRVPTO vector as previously described.^94^ For the MTDH promoter wild-type and mutant luciferase reporter plasmids, the promoter region (position -1 to -506 nt, the A in the first ATG of translation was defined as position 0) of the human *MTDH* gene was cloned and inserted into the pGL4.10 backbone. Py8119-OVA-Luc was generated as described in our previous study.^26^ Lentiviral (pLKO and pTRIPZ) and retroviral (pRVPTO) plasmids were packaged into viral particles following standard protocols. For lentivirus production, HEK293T cells were used as packaging cells and co‑transfected with the transfer plasmid together with the helper plasmids VSVG and dR8.9. For retrovirus production, H29 cells containing retroviral packaging and envelope components were transfected with the pRVPTO constructs. Viral supernatants were collected 48–72 h after transfection and filtered through a 0.45 µm membrane. Target cells were infected with viral media in the presence of 8 µg/mL polybrene and subsequently selected with puromycin and/or blasticidin to establish stable cell lines.

### Splenocyte isolation and tumor/immune cell coculture

Splenocytes were isolated as previously described.^26^ Briefly, spleens from OT-I mice were harvested into 50 ml conical tubes containing serum-free RPMI-1640 media. Spleens were smashed and filtered to remove cell debris. Red blood cells were lysed with ACK buffer (Fisher). For tumor/immune cell coculture, Py8119-OVA-Luc tumor cells, either with or without target gene knockdown, and with or without 50 nM BYL719 treatment (MCE, HY‑15244), were seeded in 6‑well plates in DMEM/F12 medium as described above for in vitro coculture. For FAM114A1 antisense oligonucleotide (ASO)-based knockdown, Py8119-OVA-Luc tumor cells were treated with 50 µM FAM114A1-targeting ASOs (ASO#1:/i2OMeU/*/i2OMeU/*/i2OMeG/*/i2OMeG/*/i2OMeC/*T*T*C*T*C*T*C*A*A*C*/i2OMeC/*/i2OMeU/*/i2OMeC/*/i2OMeU/*/i2OMeG/, ASO#2:/i2OMeU/*/i2OMeC/*/i2OMeU/*/i2OMeA/*/i2OMeG/*C*T*T*C*T*C*T*C*C*A*/i2OMeU/*/i2OMeC/*/i2OMeC/*/i2OMeA/*/i2OMeG/, ASO#3:/i2OMeC/*/i2OMeG/*/i2OMeG/*/i2OMeC/). When tumor cell cultures attained 50–75% confluency, OT‑I splenocytes were introduced at a tumor‑to‑immune cell ratio of 1:10. At the indicated time points (as described in each experiment), the cells were collected for further experiments.

### Chromatin immunoprecipitation (ChIP) and RT‒qPCR analyses

A sonication ChIP kit obtained from ABclonal (Cat# RK20258) was used according to the manufacturer’s instructions. Briefly, Py8119 cells were collected and fixed with 1% formaldehyde. The cells were then lysed with cell swelling buffer, followed by sonication. The samples were subsequently centrifuged and subjected to immunoprecipitation (IP) with an anti-E2F4 antibody (10923-1-AP, Proteintech, 5 µg) or an IgG control (provided by the kit). Each IP sample contained 15 µg of chromatin. The IP products were eluted with ChIP elution buffer, followed by decrosslinking. DNA from the samples was extracted and stored for subsequent analysis.

For RT‒qPCR analyses, total RNA from the cells specified in each experiment was isolated using an RNA extraction kit from Nanjing Vazyme Biotech Co. (Cat# RC112-01) and subsequently converted to cDNA with a SuperScript^TM^ IV kit. Real-time RT‒PCR (RT‒qPCR) was performed on a QuantStudio 5 PCR machine (Applied Biosystems) with SYBR Green qPCR Master Mix (MCE, HY-K0501A). The gene-specific primer sets were used at a final concentration of 0.2 μM. All RT‒qPCR assays were performed in duplicate in at least three independent experiments using three different cell or tissue samples.

### Flow cytometry

For tumors, samples were dissected and prepared as previously described.^26,93^ For the coculture samples, the media were removed, and the cells were digested with 0.25% trypsin-EDTA (G4011, Servicebio), collected, and washed with ice-cold DPBS (G4200, Servicebio). The cell suspensions were incubated with an antibody cocktail at a 1:200 dilution of antibody:FACS buffer (PBS + 3% BSA) for 30 min 4 °C, washed, and resuspended in FACS buffer for flow cytometry analysis. Appropriate isotype controls were employed to verify the specificity of antibody binding. For immune and tumor cell staining, the following reagents were used: DAPI (Thermo Fisher, #62248, 1:1000) or Fixable Viability Dye eFluor™ 506 (Thermo Fisher, #65‑0866‑14, 1:1000) for live/dead discrimination; PerCP‑Cy5.5 anti‑mouse CD45 (eBioscience, #45‑0451‑82); FITC, APC, and APC‑Cy7 anti‑mouse CD8a (eBioscience, #11‑0081‑82; BioLegend, #100712; BioLegend, #100714, respectively); APC‑Cy7 anti‑mouse CD3 (BioLegend, #100330); PE anti‑mouse CD137 (BioLegend, #106106); APC anti‑mouse OVA256‑264 peptide (SIINFEKL) bound to H‑2Kb (BioLegend, #141605); FITC Annexin V Apoptosis Detection Kit with 7‑AAD (BioLegend, #640922); and BD FITC BrdU Flow Kit (Fisher Scientific, #BDB559619). Gating strategies are detailed in Supplementary Figure 16. Cytometry acquisition was performed with BD FACSDiva v6 software, and analyses were conducted using FlowJo v10.

### Cell cycle synchronization and analysis

Py8119 cells at 20–30% confluence were treated with 2 mM thymidine (Beyotime, ST1704) for 16 h. The cells were then washed with PBS and cultured with fresh media for 8 h, followed by a second round of blocking with 2 mM thymidine for another 16 h. The cells were washed with PBS and released from the double thymidine block. Following release, cells were harvested at the designated time points for cell cycle and apoptosis assessment using the Beyotime Cell Cycle and Apoptosis Analysis Kit (Cat# C1052).

### Doxycycline (Dox) and anti-PD-1 in vivo treatment

OT-I female mice inoculated with Py8119-OVA-Luc with inducible FAM114A1-knockdown lentivirus were allocated into four treatment groups upon establishment of primary tumors (palpable for two consecutive weeks) and administered either Dox, anti‑PD‑1 antibody, or both agents in combination.

For Dox treatment, 2 g of Dox (Sigma, D9891) and 50 g of sucrose (MCE, HY-B1779) were dissolved in sterile water. Water containing Dox or sucrose (vehicle) alone served as the drinking water for the mice during the experimental period. Treatment with the PD‑1 antibody (BioXcell, #BP0146) was carried out in accordance with a previously published protocol.^95^ Mice were administered 200 µg of antibody intraperitoneally on days 0, 4, and 7, and subsequently once per week. Rat IgG2a (BioXcell, #BP0089) was administered according to the same regimen as a control.

### Immunoprecipitation (IP), mass spectrometry, and western blot (WB) analysis

IP experiments were performed as previously described.^94^ Briefly, cells with or without plasmid transfection (as indicated in each experiment) were collected and lysed with IP lysis buffer (20 mM Tris, pH 7.4, 0.15 M NaCl, 1 mM EDTA, 1 mM EGTA, and 1% Triton X-100) containing a complete protease inhibitor cocktail (Roche, 14493900). The samples were centrifuged, 100 µl of input mixture was added, and the remaining mixture was incubated with 5 µg of anti-FAM114A1 (Thermo Fisher, A305-742A), anti-p85α (Proteintech, 60225-1-Ig), anti-E2F4 (Proteintech, 10923-1-AP), anti-Myc (Proteintech, 16286-1-AP), or anti-GFP (MCE, HY-P80141) (specifically indicated in each experiment) antibodies overnight at 4 °C. The next day, 30 µl of protein A/G magnetic beads (Selleck, B23202) was added to each sample for another 2 h of incubation at 4 °C. The beads were washed and denatured with 1× SDS Laemmli buffer, and the samples were saved for the following assays. Uncropped Western blot images are provided in Supplementary Fig. 17.

For mass spectrometry to identify FAM114A1-binding proteins, the samples were separated by SDS–PAGE, followed by staining of the gels with Coomassie Brilliant Blue. The target proteins in the gel were excised, and in-gel digestion was performed to obtain peptide solutions. The peptides were subjected to nano-HPLC‒MS/MS analysis. To identify target proteins, tandem mass spectra were extracted and processed with Proteome Discoverer software (Thermo Fisher Scientific, version 3.1). Tandem mass spectra were searched against a human protein database in which the digestion enzyme trypsin was used.

For WB, the cells were lysed with IP lysis buffer, and the cytoplasmic and nuclear fractions were extracted via a nuclear and cytoplasmic protein extraction kit (Proteintech, PK10014). The samples were resolved via SDS‒PAGE and immunoblotted according to standard protocols. The samples were blotted with the following antibodies: anti-Cas9 (CST, #14697, dilution 1:1000), anti-MS2 (Sigma, ZRB2123, dilution 1:1000), anti-ovalbumin (Proteintech, 67614-1-Ig, dilution 1:1000), anti-β-actin (Proteintech, 66009-1-Ig, dilution 1:5000), anti-FAM114A1 (Proteintech, 67926-1-Ig, dilution 1:1000), anti-phosphorylated AKT (S473) (Proteintech, 66444-1-Ig, dilution 1:1000), anti-AKT (CST, #4691, dilution 1:1000), anti-p85α (Proteintech, 60225-1-Ig, dilution 1:1000), anti-Myc-tag (MCE, HY-P80232, dilution 1:1000), anti-p110α (Proteintech, 67071-1-Ig, dilution 1:1000), anti-E2F4 (Proteintech, 10923-1-AP, dilution 1:1000), anti-GFP (MCE, HY-P80141, dilution 1:1000), and anti-Vinculin (Proteintech, 26520-1).

### CRISPRa screening

Py8119-OVA cells were transduced with viruses expressing dCas9-VP64 (Addgene#61425) and MS2-P65-HSF1 (Addgene#89308) sequentially. The cells were selected with blasticidin or hygromycin to generate a stable cell line. The cells were then transduced with a mouse CRISPR activation library (SAM) at an MOI of 0.1 and coverage of 500, followed by selection with puromycin to pick positive cells. The cells were cocultured with splenocytes from OT-I mice at a ratio of 1:10 (tumor:immune) for 72 h. Non-cocultured cells served as a negative control. The cells were collected and stained with DAPI to sort living cells. The genomic DNA of living cells was extracted for sgRNA amplification and next-generation sequencing.

### Protein purification and in vitro droplet assay

Plasmids containing Strep-GFP-E2F4 or GST-FAM114A1 were transformed into *E. coli* BL21 cells. After induction with isopropyl-β-D-thiogalactoside (IPTG), the bacterial pellets were collected, resuspended in lysis buffer (50 mM Tris-HCl, pH 7.5, 500 mM NaCl, 1 mM dithiothreitol (DTT), 1 mM phenylmethanesulfonyl fluoride (PMSF), and 1% Triton X-100), and sonicated. After centrifugation, the Strep-tagged recombinant protein was purified via Strep-Tactin Superflow beads (IBA Life Sciences). The GST and GST-fusion proteins were purified via glutathione Sepharose beads. The eluted proteins were concentrated via Amicon Ultra Centrifugal Filters (Millipore). The protein concentrations were measured via a Bradford protein quantification kit (Vazyme).

The purified GST, GST-FAM114A1, and E2F4-GFPs were diluted to various concentrations in buffer containing 50 mM Tris-HCl (pH 7.5), 10% glycerol, and 1 mM DTT with the indicated salt concentrations. The protein mixture was loaded onto a glass slide, covered with a coverslip, and imaged via a fluorescence microscope.

### Live-cell imaging and fluorescence recovery after photobleaching (FRAP) assay

Live imaging and FRAP assays were performed as previously described.^96^ In detail, cells transfected with the E2F4-GFP plasmid were seeded on a 35 mm glass-bottomed cell culture dish and examined under a Zeiss LSM 900 confocal microscope. During image acquisition, the cells were maintained in an equilibrated observation chamber at 37 °C with 5% CO_2_. Images were captured at 2-s intervals and analyzed via ImageJ to identify fusion events.

FRAP experiments were performed via a Zeiss LSM 900 confocal microscope. For FRAP analysis of living cells, cells grown on 35 mm glass-bottomed cell culture dishes were transfected with E2F4-GFP for 24 h. The E2F4-GFP puncta were then photobleached with 15% laser power for 500 ms via a 488 nm laser. Time-lapse images were acquired for the indicated times at 2-s intervals after bleaching. For FRAP analysis in vitro, GFP-E2F4 droplets on a glass slide were covered with a coverslip and photobleached. The fluorescence recovery data were corrected for photobleaching by reference to unbleached regions and subsequently normalized to the pre‑bleach intensities of the regions of interest (ROIs). Curve fitting was performed using a single‑exponential model in GraphPad Prism 8.

### Statistical analysis

Animals were excluded solely in cases of death or euthanasia in accordance with institutional IACUC guidelines. The sample size was not predetermined by statistical methods. Data acquisition and analysis were conducted without investigator blinding. In vivo studies involved randomization of animals and treatment as described for each experimental group. In vitro assays were performed with all samples analyzed equally and repeated at least three times to ensure reproducibility. Statistical methods are detailed in the figure captions. Results are expressed as means ± SEMs, and analyses were carried out using GraphPad Prism (version 8).

## Supplementary information

Supplementary Figures

Supplementary Table 1

Supplementary Table 2

Supplementary Table 3

Supplementary Table 4

Supplementary Table 5

Supplementary Table 6

## Data Availability

The sequencing data generated during this study have been deposited in the Gene Expression Omnibus (GEO) at GSE295176. The TNBC patient expression profile data analyzed in this study were obtained from GEO at GSE173839, GSE194040, and GSE169246; from the SRA at SRP157974; from the BioProject at PRJNA558949; and from the TCGA at https://xenabrowser.net/datapages/. For NCT03197389 (EGAD00001006608), the data were requested from https://lambrechtslab.sites.vib.be/en/single-cell.
