# Supplementary material for: Family with sequence similarity 114 member A1 orchestrates immune evasion in triple-negative breast cancer
Source: Signal Transduct Target Ther. 2025 Nov 18;10:373. doi: 10.1038/s41392-025-02472-9 (PMC12624017; doi:10.1038/s41392-025-02472-9)
Supplement: Supplementary file 1 — Supplementary Figures [file 41392_2025_2472_MOESM1_ESM.docx]

**Supplementary Materials for**

**Family With Sequence Similarity 114 Member A1 orchestrates immune evasion in triple-negative breast cancer**

Wenhao Zhang, Yanzhi Gai, Mengxue Qiao, Michelle Rowicki, Yong Wei, Xiang Hang, Zhengkai Wei, He Yang, Xifu Ye, Hang Ju, Yi Lu, Yibin Kang, Minhong Shen

Correspondence to：[minhong@fudan.edu.cn; ykang@princeton.edu](mailto:minhong@fudan.edu.cn; ykang@princeton.edu); and [yilu_tju@tongji.edu.cn](mailto:yilu_tju@tongji.edu.cn)

**This PDF file includes:**

Supplementary Figures. 1 to 17

**Other Supplementary Materials for this manuscript include the following:**

Supplementary Table 1. Table showing TNBC patient cohorts and corresponding FAM114A1 expression.

Supplementary Table 2. Table showing 109 TNBC patient samples and their FAM114A1 expression.

Supplementary Table 3. Table showing proteins identified by FAM114A1 co-IP mass spectrometry analysis.

Supplementary Table 4. Table showing the predicted binding affinities between the E2F4 and TFDP/RB proteins.

Supplementary Table 5. Table showing regression analysis between *FAM114A1* expression and tumor characteristics.

Supplementary Table 6. Table showing the FAM114A1 signature gene list.

**Supplementary Fig. 1**

**Supplementary Fig. 1. Tumor/Immune coculture based CRISPRa screening.** **a**, Py8119 cells were transduced with lentivirus to stably express SAM CRISPRa screening components and ovalbumin. The expressions of dCas9, P65/HSF1, and ovalbumin were validated with western blotting. OVA, ovalbumin. **b**, Indicated cells were cocultured with splenocytes isolated from OT-I mice at tumor cell:immune cell=1:10. Twenty-four hours after coculture, the surface presentation of ovalbumin on tumor cells was measured (left panel) and quantified (right panel) with flow cytometry. **c**, Indicated cells were cocultured with OT-I splenocytes as in (**b**). Viable cells were quantified after 24 hours. **d**, Schematic diagram of CRISPRa screening to identify genes that promote immune evasion. **e,** FAM114A1 guide RNA sequences used in CRISPRa screening system. **f,** Western blotting examination of FAM114A1 protein levels in Py8119 tumor cells with (gRNAs) and without (vector) FAM114A1 guide RNA sequences transduced. β-actin served as internal control (left panel). The expression levels have been quantified (right panel). **g**, Py8119 cells labeled with ovalbumin and firefly luciferase (Py8119-OVA-Luc) were used to generate endogenous FAM114A1 stable knockdown cell lines. Cells with stable FAM114A1 knockdown (KD#1 and KD#2) and corresponding control (shCoo2) were cocultured with OT-I splenocytes as above. Viable cells were quantified with luciferase assay after 24 hours. The data represent the means ± SEMs. P values were determined by one-way ANOVA (**b**, **g**) or two-tailed Student’s *t* test (**c**).

**Supplementary Fig. 2**

**Supplementary Fig. 2. FAM114A1 knockdown enhances CD8^+^ T-cell infiltration. a**, UMAPs present cell populations from All, control (shCoo2), and FAM114A1 knockdown (FAM114A1-KD) mouse tumor groups as in Fig. 1f. Tumor cells are highlighted with a red dished circle in each group, and the expression of FAM114A1 is shown with colored scale. **b,** UMAP showing T cell subpopulations in All, control (shCoo2), and FAM114A1 knockdown (FAM114A1-KD) tumor groups as in Fig. 1f. CD8^+^ T cell populations are highlighted with blue dashed circles (left panel). Average cell type proportions in shCoo2 and FAM114A1-KD are shown (right panel). Red start indicates CD8^+^ T cell population. **c**, IHC staining of CD8^+^ T cells in tumors from Fig. 1e. Representative images are shown. Bar, 50 µm. **d**, Number of CD8^+^ T cells per filed was quantified. n=6 tumors per group. The data represent the means ± SEMs. P values were determined by one-way ANOVA test (**d**).

**Supplementary Fig. 3**

**Supplementary Fig. 3. FAM114A1 high expression results in less CD8^+^ T-cell infiltration and activation in TNBC patients. a-c**, Single cell RNA sequencing data of TNBC patients (NCT03197389) was extracted and analyzed. UMAPs showing populations of indicated cells. Red or blue dashed circles highlight T, CD8^+^ T, or activated CD8^+^ T populations respectively. Average cell type proportions in high- and low-*FAM114A1* patients are also shown. *FAM114A1* high (n=7) and low (n=6) patients were stratified on the basis of median expression. Red starts indicate T cell (**a**), CD8^+^ T cell (**b**), and activated CD8^+^ T cell (**c**) populations respectively.

**Supplementary Fig. 4**

**Supplementary Fig. 4. FAM114A1 is involved in the PI3K and antigen presentation pathways. a,** Tumor cells were extracted from scFFPE-Seq data as in Fig. 1f. Enrichment analyses indicated pathways that were enriched in control (shCoo2) and FAM114A1 knockdown (KD) mouse tumors. **b,** Heatmap showing antigen presentation signature and activated PI3K/AKT marker genes in tumor cells from shCoo2 and FAM114A1-KD groups. **c,** Bulk RNA sequencing data were extracted from FUSCC dataset (SRP157974). Patients were classified into high (top 20 patients)- and low (bottom 20 patients)-*FAM114A1* expression groups. Pathways enriched in *FAM114A1* high and low patients are shown.

**Supplementary Fig. 5**


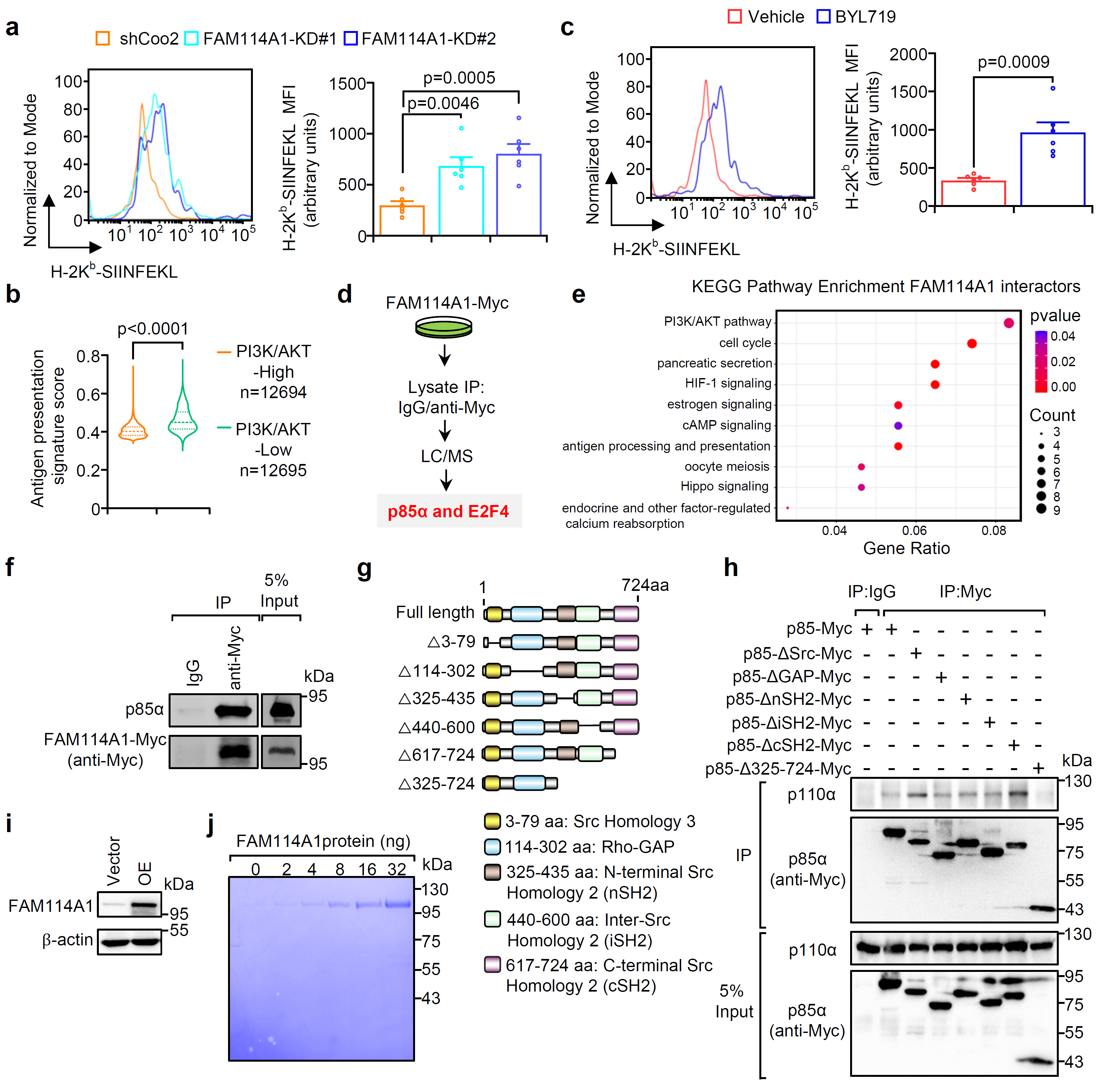


**Supplementary Fig. 5. PI3K/AKT activation inhibits antigen presentation in tumor cells. a**, Py8119-OVA-Luc cells with (FAM114A1-KD#1 and KD#2) or without (shCoo2) FAM114A1 knockdown were cocultured with splenocytes isolated from OT-I mice. Two hours after coculture, ovalbumin presentation on tumor cells were measured (left panel) and quantified (right panel) with flow cytometry. **b**, Single cell RNA sequencing data of cancer cells was extracted from TNBC patients (NCT03197389). PI3K/AKT activation scores were calculated and the cells were stratified into high- and low-PI3K/AKT activation groups on the basis of median. Antigen presentation signature scores were calculated in each cell. **c**, Py8119-OVA-Luc cells were treated with 50 nM of BYL719 or DMSO (Vehicle) for 4 hours. Cells were released from the treatment and were then cocultured with OT-I splenocytes for 2 hours. Cells were collected and ovalbumin presentation was measured and quantified with flow cytometry. **d**, Schematic diagram of coimmunoprecipitation (co-IP) followed by mass spectrometry to identify FAM114A1 interacting proteins. **e,** FAM114A1 interacting proteins identified by our co-IP mass spectrometry assay were selected for pathway enrichment analysis. **f**, 293T cells were co-transfected with plasmids to overexpress FAM114A1-Myc and p85α. The cells were collected for co-IP after 24 hours and samples were analyzed with western blotting. **g**, Schematic diagram of domain deletion mutants of p85α. **h**, 293T cells were co-transfected with indicated plasmids. Twenty-four hours after transfection, the cells were lysed and subjected to co-IP analysis. The samples were then analyzed with western blotting to detect the interactions. **i**, Py8119 cells with stable FAM114A1 expression were generated. The cells with (OE) or without (Vector) FAM114A1 overexpression were validated with western blotting. **j**, Coomassie blue staining of the recombinant FAM114A1 protein. The data represent the means ± SEMs. P values were determined by one-way ANOVA (**a**) or two-tailed Student’s *t* test (**b, c**).

**Supplementary Fig. 6**

**Supplementary Fig. 6. FAM114A1 knockdown increases E2F4 condensate formation.** **a**, 293T cells were co-transfected with plasmids to overexpress FAM114A1-Myc and E2F4-GFP. Cells were collected for co-IP after 24 hours and samples were analyzed with western blotting. **b, c**, 4TO7 cells with (KD#1 and KD#2) and without (shCoo2) FAM114A1 knockdown were fixed and subjected to immunofluorescence (IF) staining with the anti-E2F4 antibody. DNA was visualized with DAPI (**b**). Percentage of cells with E2F4 condensates was quantified (**c**). Bar, 10 µm. **d**, Py8119 cells transfected with E2F4-GFP were treated with 5% Hex for 1 minute and subjected to confocal imaging. DNA was visualized with DAPI. Hex, 1,6-hexanediol. Bar, 10 µm. **e**, Py8119 cells transfected with E2F4-Myc were subjected to IF staining with the anti-Myc antibody and followed by confocal imaging. DNA was visualized with DAPI. Bar, 10 µm. **f**, Schematic diagram of domain deletion mutants of E2F4. The FAM114A1 binding capacity is indicated. **g**, IUPred assigned scores of disordered tendencies between 0 and 1 to E2F4 (a score of more than 0.5 indicates disordered). **h**, Py8119 cells transfected with indicated E2F4 mutants were collected for western blotting. Vinculin served as internal control. **i**, Percentage of cells with E2F4 condensates was quantified in Py8119 cells transfected with indicated E2F4-GFP mutants. The data represent the means ± SEMs. P values were determined by one-way ANOVA test (**c**).

**Supplementary Fig. 7**

**Supplementary Fig. 7. E2F2 antibody specifically recognizes endogenous E2F4. a,** Py8119 tumor cells with (E2F4-KD) and without (shCoo2) endogenous E2F4 knockdown were subjected to immunofluorescence (IF) staining with the anti-E2F4 antibody. **b,** TNBC patient samples were subjected to IF staining with the anti-E2F4 antibody or IgG isotype control. Bar, 10 µm.

**Supplementary Fig. 8**


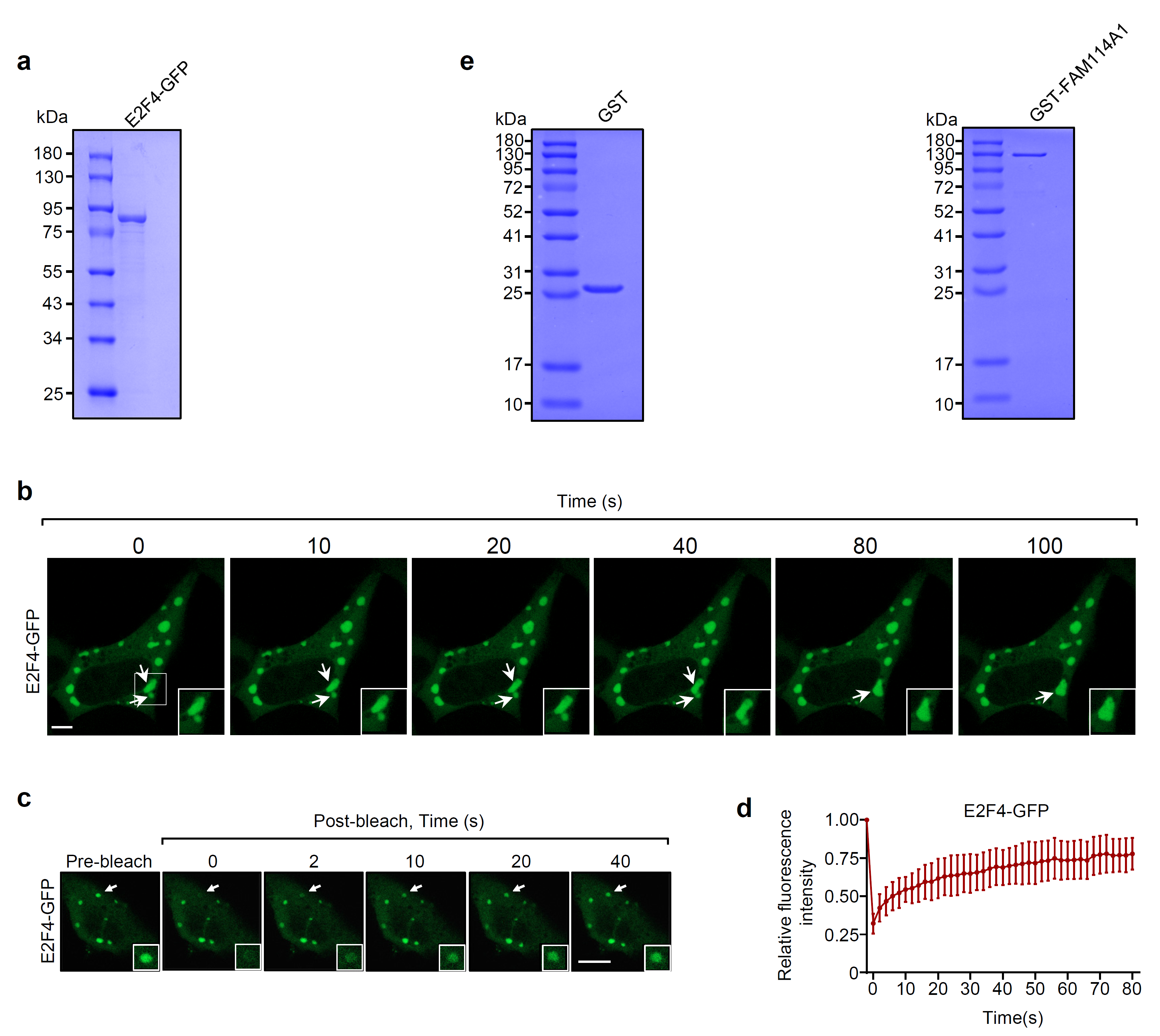


**Supplementary Fig. 8. E2F4 condensates exhibit liquid-like properties.** **a**, Coomassie blue staining of E2F4-GFP recombinant protein used for droplet formation assays. **b**, MDA-MB-231 breast cancer cells were transfected with E2F4-GFP. Live-cell imaging of E2F4-GFP condensates. The arrows indicate representative E2F4 condensates that fused. Bar, 10 µm. **c**, MDA-MB-231 breast cancer cells were transfected with E2F4-GFP. E2F4 condensates were subjected to FRAP assay. The arrows indicate the condensate upon photobleaching. The condensates at different timepoints after photobleaching are enlarged and shown at right bottom corner of each representative image. Bar, 10 µm. **d**, Average values for the FRAP data are shown. **e**, Coomassie blue staining of GST and GST-FAM114A1 recombinant proteins used for droplet formation assays in Fig. 4g. The data represent the means ± SEMs.

**Supplementary Fig. 9**

**Supplementary Fig. 9. E2F4 promotes MTDH expression. a,** Schematic diagram of *MTDH* promoter region in firefly luciferase reporter. 11 putative motif clusters that bind to E2F4 are shown. **b**, 11 mutant *MTDH* luciferase reporters with each motif cluster depletion were generated. Luciferase reporters containing mutant or wild-type (FL) *MTDH* promoter region were co-transfected with E2F4 and Renilla luciferase plasmids into Py8119 cells. Firefly luciferase activity was measured and normalized with Renilla luciferase activity. **c, d**, 4TO7 cells with (E2F4-KD#1 and KD#2) and without (shCoo2) stable E2F4 knockdown were collected to perform RT-qPCR or western blotting to detect E2F4 or MTDH mRNA levels (**c**) or protein levels (**d**) respectively. **e**, Py8119 cells with (E2F4-KD) or without (shCoo2) stable E2F4 knockdown were rescued with wild-type or mutant E2F4 plasmid as indicated. The cells were collected for RT-qPCR to examine the expression of *Mtdh*. **f**, WT *MTDH* luciferase reporter together with Renilla luciferase plasmids were co-transfected with indicated E2F4 plasmids into Py8119 cells. Twenty-four hours after transfection, normalized firefly luciferase signal was determined. The data represent means ± SEMs. P values were determined by one-way ANOVA test.

**Supplementary Fig. 10**

**Supplementary Fig. 10. E2F4 condensates formation is negatively correlated with its nuclear translocation.** **a**, Full length or indicated E2F4-GFP mutants were transfected into Py8119 cells. Percentage of GFP signal in nuclei was quantified. **b**, Cytosol and nuclear fractions of the cells from (**a**) were extracted and subjected to western blotting analysis. The ratio of nuclear E2F4-GFP was quantified. Vinculin and Lamin B1 served as internal controls for cytosol and nuclear fractions respectively. **c, d**, 4TO7 cells with (KD#1 and KD#2) or without (shCoo2) stable FAM114A1 knockdown were used to extract cytosol or nuclear proteins. The expressions of E2F4 and FAM114A1 in cytosol and nuclear were examined with western blotting (**c**). The cells were subjected to RNA and protein extraction. The mRNA levels and protein levels of FAM114A1 and MTDH were examined with RT-qPCR and western blotting respectively (**d**). **e, f,** Indicated cells were cocultured with splenocytes isolated from OT-I mice at tumor cell:immune cell=1:10. Four hours after coculture, the surface presentation of ovalbumin on tumor cells was measured (left panel) and quantified (right panel) with flow cytometry. shCoo2: Py8119-OVA-Luc PLKO-shCoo2; FAM114A1-KD: Py8119-OVA-Luc PLKO-shFAM114A1; FAM114A1-KD+MTDH-OE: Py8119-OVA-Luc PLKO-shFAM114A1 with MTDH overexpressed; E2F4-KD: Py8119-OVA-Luc PLKO-shE2F4; E2F4-KD+MTDH-OE: Py8119-OVA-Luc PLKO-shE2F4 with MTDH overexpressed. n=5 replicates per group. The data represent means ± SEMs. P values were determined by one-way ANOVA test.

**Supplementary Fig. 11**

**Supplementary Fig. 11. FAM114A1-knockdown induces cell proliferation and viability defects. a, b,** Py8119-OVA-Luc tumor cells with FAM114A1 knockdown and the corresponding control were employed for tumor sphere assay. 2x10^4^ cells were seeded for each group. Five days after culture, spheres were imaged (**a**), and the number and size of the spheres were quantified (**b**). n=3 replicates per group. The size of 20 randomly picked spheres were measured in **b**. bar, 50 µm. **c,** Py8119 tumor cells with and without FAM114A1 knockdown were injected into Nude mice orthotopically. Tumors from indicated groups were collected for IHC staining with anti-Ki67 and cleaved caspase 3 (CC-3) antibodies. Representative images are shown. n=7 and 8 mice for control (shCoo2) and FAM114A1 knockdown (KD) group respectively. Bar, 50 µm. shCoo2: Py8119-OVA-Luc PLKO-shCoo2; KD#1: Py8119-OVA-Luc PLKO-shFAM114A1#1; KD#2: Py8119-OVA-Luc PLKO-shFAM114A1#2. The data represent means ± SEMs. P values were determined by one-way ANOVA test (**b**).

**Supplementary Fig. 12**

**Supplementary Fig. 12. FAM114A1-induced E2F4 condensate-formation inhibition and antigen-presentation suppression are independent of its cell cycle regulation. a,** Py8119 cells were synchronized with a double thymidine block. The cells at indicated phases were subjected to immunofluorescence staining with the anti-E2F4 antibody. DNA was visualized with DAPI. Bar, 10 µm. **b,** Py8119 cells at indicated time points after release were collected for western blotting analysis. The expressions of MTDH and FAM114A1 were examined. β-actin served as internal control. asy.: asynchronized.

**Supplementary Fig. 13**

**Supplementary Fig. 13. FAM114A1-mediated immunosuppression is critical for its tumor-promoting function. a,** Py8119-OVA-Luc tumor cells with and without FAM114A1 knockdown were subjected to tumor sphere assay. Five days after culture, 1x10^6^  OT-I splenocytes were added into the coculture groups. Fouty-eight hours after coculture, the viable tumor cells were determined by luciferase assay (left panel). The ratio of viable tumor cells was normalized with their corresponding non-coculture groups to rule out the proliferation and viability defects (right panel). n=3 replicates per group. shCoo2: Py8119-OVA-Luc PLKO-shCoo2; KD: Py8119-OVA-Luc PLKO-shFAM114A1. **b,** OT-I female mice were pretreated with 125 μg/mouse of anti-CD8 antibody or isotype control every two days for one week. Py8119-OVA-Luc tumor cells with/without FAM114A1 knockdown were orthotopically injected into the mice, and the mice were continually treated with 125 μg/mouse of anti-CD8 antibody or isotype control twice per week to the end of the experiment. At the endpoint, tumors were collected and CD8^+^ T-cell depletion efficiency was validated with flow cytometry analysis. The data represent means ± SEMs. P values were determined by two-tailed Student’s *t* test.

**Supplementary Fig. 14**


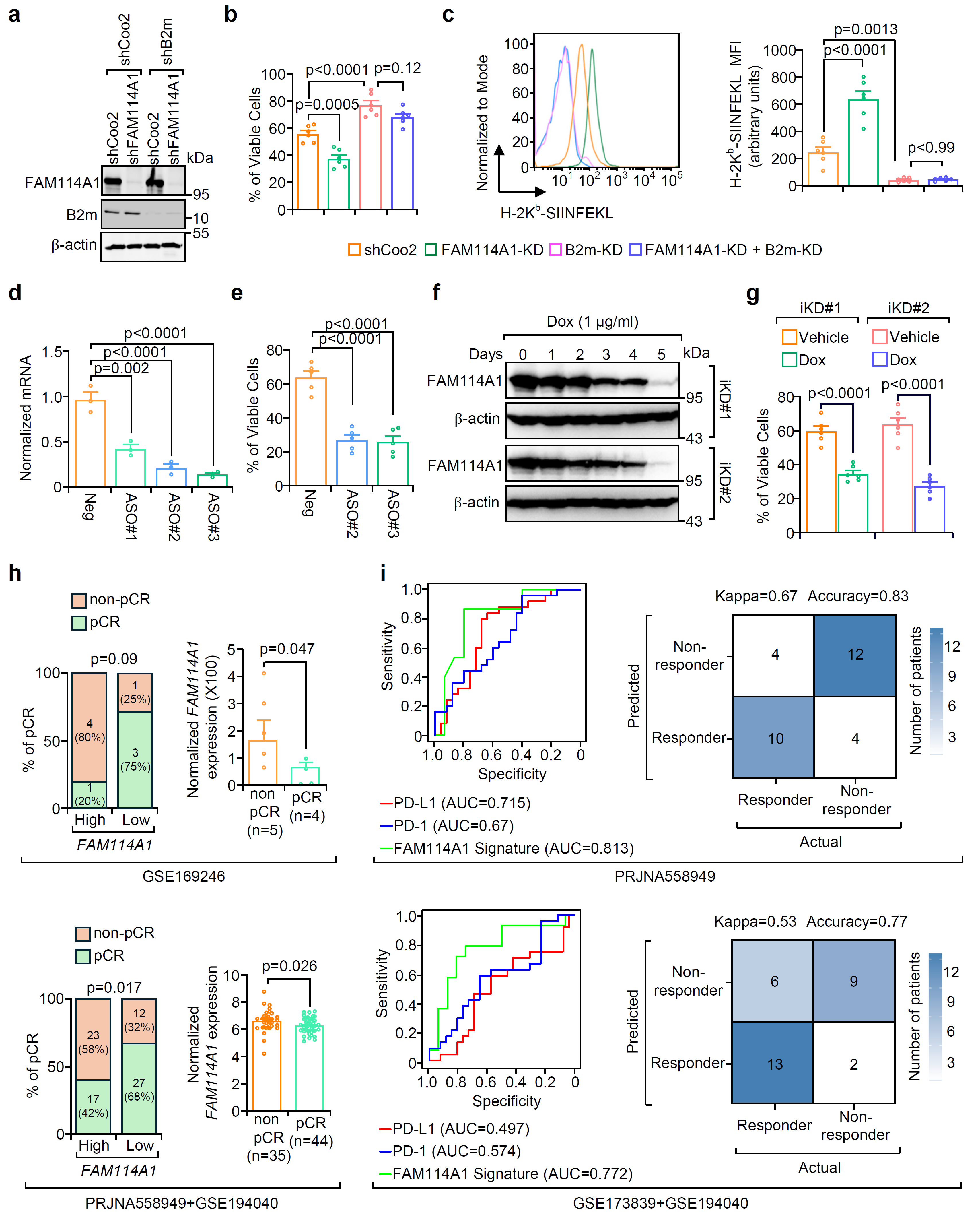


**Supplementary Fig. 14. FAM114A1 promotes TNBC immune evasion via antigen presentation suppression. a-c,** Py8119-OVA-Luc cells with FAM114A1 or B2m knockdown alone or in combination were generated. The knockdown efficiency was validated with western blotting (**a**). The cells were cocultured with OT-I splenocytes and viable tumor cells were quantified with luciferase assay after 24 hours (**b**). Similar coculture as in (**b**) was performed. Tumor cells were collected 2 hours after coculture, and ovalbumin antigen presentation was measured and quantified with flow cytometry (**c**). **d,** Py8119-OVA-Luc tumor cells were treated with 50 µM of FAM114A1-targeting ASO (ASO#1, ASO#2, ASO#3) or control ASO (Neg) for 48 hours. The cells were collected and mRNA levels of FAM114A1 were determined by RT-qPCR. **e,** Py8119-OVA-Luc tumor cells were treated with indicated ASO for 48 hours. OT-I splenocytes were then added to perform coculture assay. Viable cells were quantified with luciferase assay after 24 hours. **f**, Two Py8119-OVA-Luc cell lines (iKD#1 and iKD#2) with FAM114A1 inducible knockdown were generated. The FAM114A1 knockdown efficiency upon doxycycline (Dox) treatment was validated with western blotting. **g**, iKD#1 and iKD#2 cells were treated with 1 µg/ml of Dox for 5 days. The cells were then subjected to coculture assays with OT-I splenocytes. Viable cells were quantified after 24 hours. **h**, TNBC immunotherapy response status and *FAM114A1* expression were extracted from patient cohorts of GSE169246, GSE194040, or PRJNA558949 (NCT02489448). Patients were stratified into high- and low-*FAM114A1* groups on the basis of median expression. The percentages of pathological complete response (pCR) and non-pCR patients are shown in both *FAM114A1* high and low patients (left of each panel). The expressions of *FAM114A1* in pCR and non-pCR patients are shown (right of each panel). **i**, Receiver Operating Characteristic (ROC) curves (left) and confusion matrix (right) showing the predictive performance of the FAM114A1 signature in PRJNA558949 (NCT02489448) and I-SPY2 TNBC patient cohorts (GSE173839 and GSE194040). The data represent means ± SEMs. P values were determined by one-way ANOVA (**b, c, d, e, g**), Chi-Square (**h,** left of each panel), and two-tailed Student’s *t* test (**h,** right of each panel).

**Supplementary Fig. 15**

**Supplementary Fig. 15. Schematic diagram of working model.** FAM114A1 blocks p85α and p110α interaction to activate the PI3K/AKT signaling. Meanwhile, it prevents E2F4 condensate formation and enhances the binding between E2F4 and TFDP/RB to facilitate E2F4 nuclear translocation and consequent MTDH expression. FAM114A1-mediated PI3K/AKT and E2F4/MTDH activation promotes cell proliferation and survival, as well as inhibits tumor antigen presentation. Based on this mechanism, FAM114A1-Signature has been developed, which can facilitate recognizing TNBC patients who respond to immune checkpoint blockade therapy (ICB). FAM114A1-targeting in non-responders may sensitize these patients to ICB.

**Supplementary Fig. 16**

**Supplementary Fig. 16. Gating strategies for flow cytometry analysis.**

**Supplementary Fig. 17**


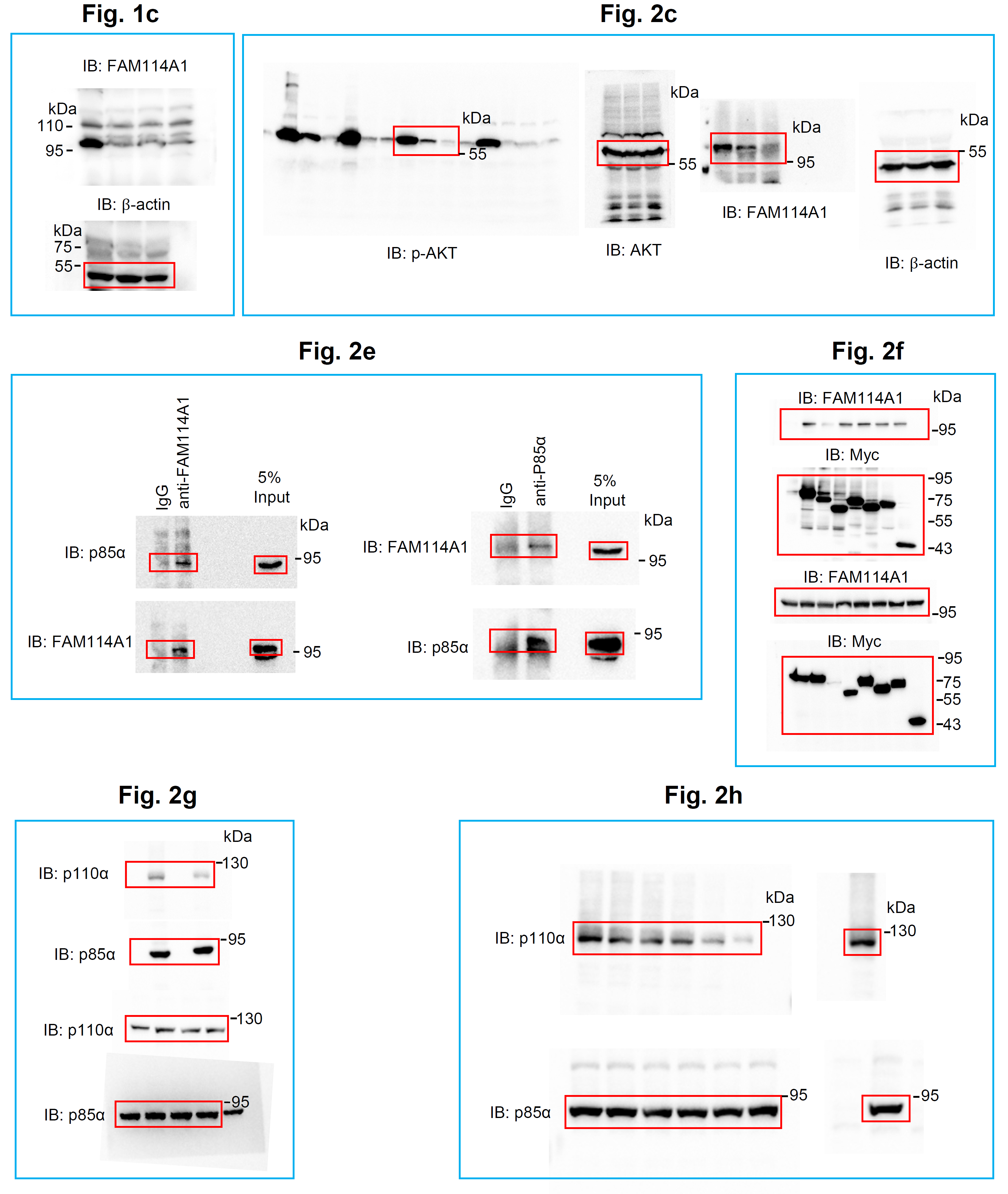


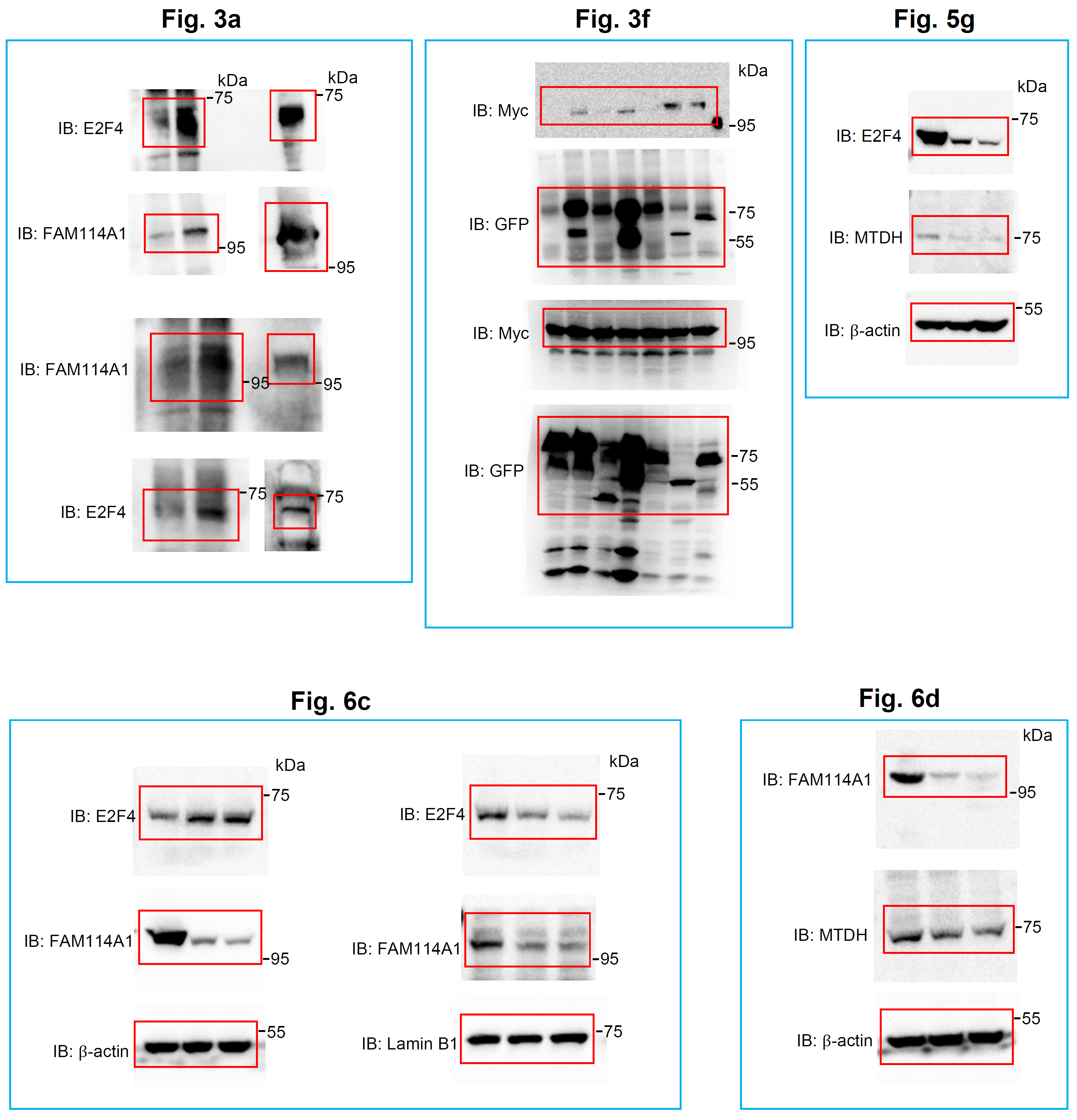


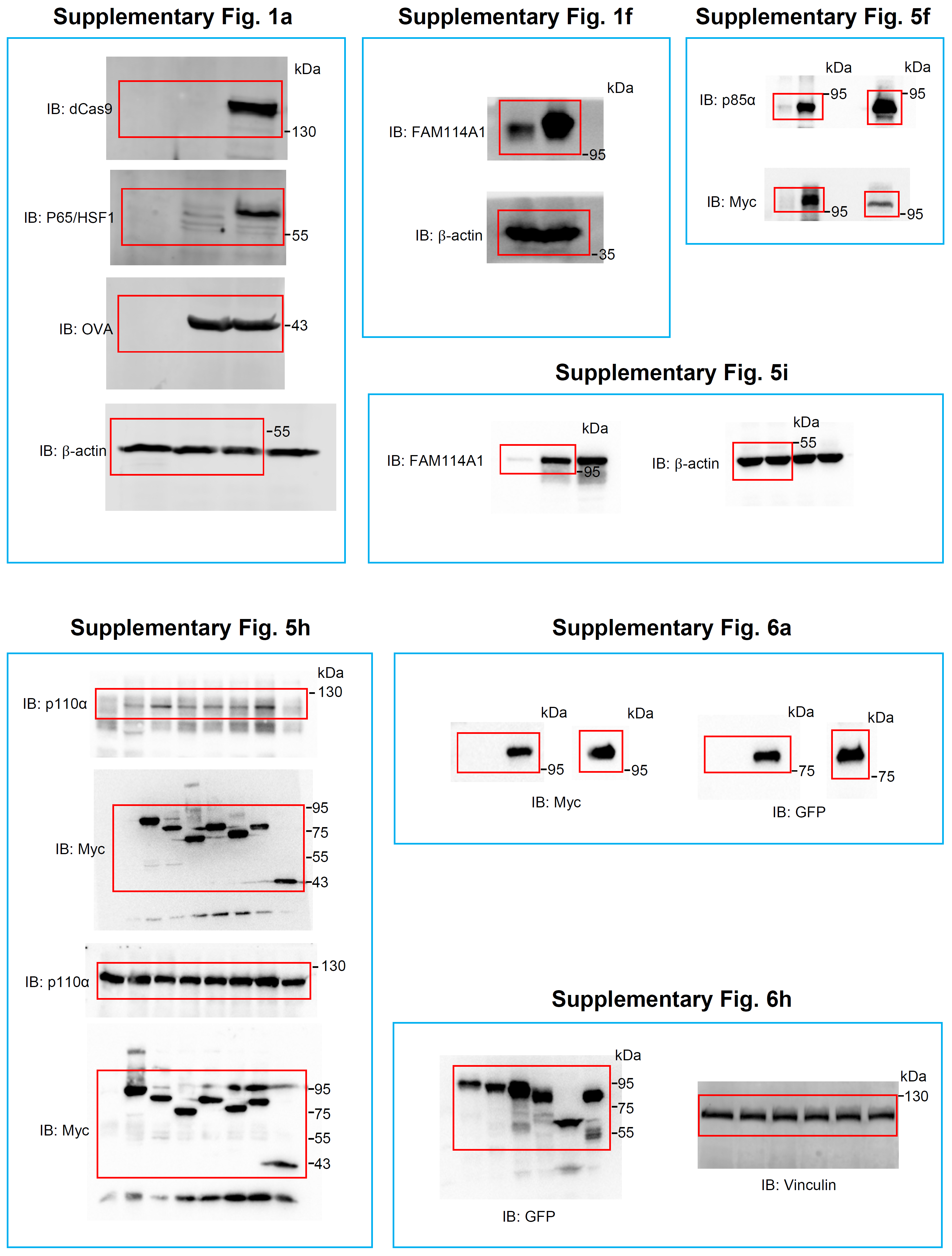


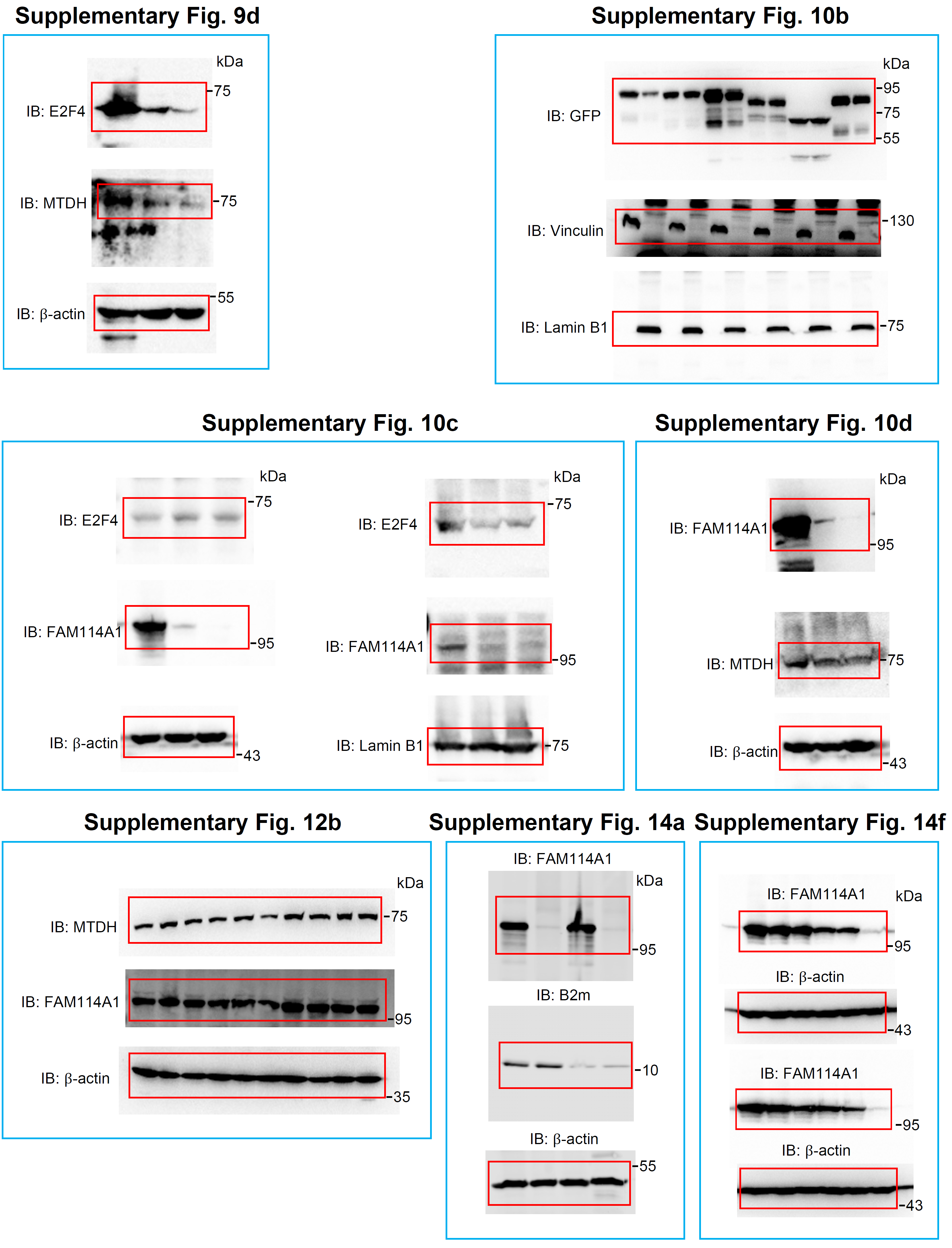


**Supplementary Fig. 17. Uncropped films of Western blots.**
